# Community mobilisation approaches to preventing adolescent multiple risk behaviour: a realist review

**DOI:** 10.1186/s13643-024-02450-2

**Published:** 2024-02-26

**Authors:** Laura Tinner, Claire Kelly, Deborah Caldwell, Rona Campbell

**Affiliations:** Population Health Sciences, Bristol Medical School, Canynge Hall, University of Bristol, Bristol, BS8 2PL UK

**Keywords:** Community mobilisation, Adolescence, Risk behaviour, Realist review, Complex interventions, Inequalities

## Abstract

**Background:**

Adolescent multiple risk behaviour (MRB) is a global health issue. Most interventions have focused on the proximal causes of adolescent MRB such as peer or family influence, with systematic reviews reporting mixed evidence of effectiveness. There is increasing recognition that community mobilisation approaches could be beneficial for adolescent health. There are gaps in the current literature, theory and implementation that would benefit from a realist approach. We use a theory-driven evidence synthesis to assess how and why community mobilisation interventions work/do not work to prevent adolescent MRB and in what contexts.

**Methods:**

This realist review used a six-stage iterative process, guided by the RAMESES framework. We systematically searched PubMed, MEDLINE, PsycINFO, Web of Science, CINAHL and Sociological Abstracts, from their inception to 2021. Studies were screened for relevance to the programme theory, assessed for rigour and included based on a priori criteria. Two independent reviewers selected, screened and extracted data from included studies. A realist logic of analysis was used to develop context-mechanism-outcome configurations that contributed to our programme theory.

**Findings:**

We reviewed 35 documents describing 22 separate community mobilisation intervention studies. Most studies (*n* = 17) had a quality assessment score of three or four (out of four). We analysed the studies in relation to three middle range theories. To uphold our theory that these interventions work by creating a social environment where adolescents are less likely to engage in MRB, interventions should: (1) embed a framework of guiding principles throughout the community, (2) establish community readiness with population data and (3) ensure a diverse coalition with the support of intervention champions. Mechanisms such as empowerment through coalition ownership over the delivery of the intervention, cohesion across the community and motivation to work collaboratively to improve adolescent health are triggered to achieve social environment shifts. However, certain contexts (e.g. limited funding) restrict intervention success as these mechanisms are not fired.

**Conclusions:**

For community mobilisation interventions to reduce adolescent MRB, the coalitions within them must seek to alter the social environment in which these behaviours occur. Mechanisms including empowerment, cohesion and motivation lead to this shift, but only under certain contexts.

**Systematic review registration:**

PROSPERO CRD42020205342

**Supplementary Information:**

The online version contains supplementary material available at 10.1186/s13643-024-02450-2.

## Background

Health risk behaviours including hazardous alcohol consumption, tobacco smoking, antisocial behaviour, risky sexual behaviour and physical inactivity are global health issues and are often initiated and become habitual in adolescence [1, 2]. Adolescents who engage in one risk behaviour are likely to engage in others [3, 4], leading to an increased public health focus on multiple risk behaviour (MRB), which is defined by the co-occurrence of two or more risk behaviours directly or indirectly related to health [5, 6]. MRB can result in many deleterious health and social effects later in life, such as unemployment [7], poor educational attainment [8], obesity and mental health issues [9], cancers and premature mortality [8, 10]. This has, therefore, led to public health strategies that address multiple as opposed to single behaviours [11].

Most interventions addressing adolescent MRB have focused on the proximal causes such as peer or family influence, rather than targeting the wider environmental, social or structural context [12]. For instance, two Cochrane systematic reviews have assessed the impact of individual, family and school-level interventions on adolescent multiple risk behaviour [11, 13]. One of those reviews found mixed evidence, concluding that school-based universal interventions are potentially effective in ‘preventing engagement in tobacco use, alcohol use, illicit drug use and antisocial behaviour, and in improving physical activity among young people, but not in preventing other risk behaviours’ [13]. The authors highlighted that there was no strong evidence of benefit for family‐level or individual‐level interventions across the risk behaviour outcomes investigated [13]. The interventions included in this review were predominantly educational programmes. The effectiveness and equity of these ‘downstream’ interventions have been questioned [14] because health risk behaviours rarely have a single cause and occur in complex socio-cultural contexts [15]. As such, there is increasing recognition that structural changes that extend beyond individually focused educational programmes could be beneficial for adolescent health [15, 16].

### Community mobilisation interventions

Recognition that decisions about health risk behaviours are made within a broad social context has led to the development and implementation of community-engagement interventions [17]. There is a range of community-engagement public health intervention types, which vary in the extent to which they emphasise community involvement in determining and delivering the programmes [17]. ‘Community mobilisation’ interventions are one such intervention type that work to engage community members to ‘take action towards achieving a common goal’ [18]. They have gained traction as a strategy for addressing complex and multifaceted problems [19]. Community mobilisation is a collaborative public health effort that is defined by the inclusion of a community coalition made up of diverse stakeholders (such as schools, businesses, residents, youth groups, emergency services and religious leaders) [12]. These stakeholders critically analyse the root causes of local problems, identify an array of potential solutions, develop multi-sector partnerships and implement multi-component strategies for creating local change and more health-promoting environments [15].

**Fig. 1:**
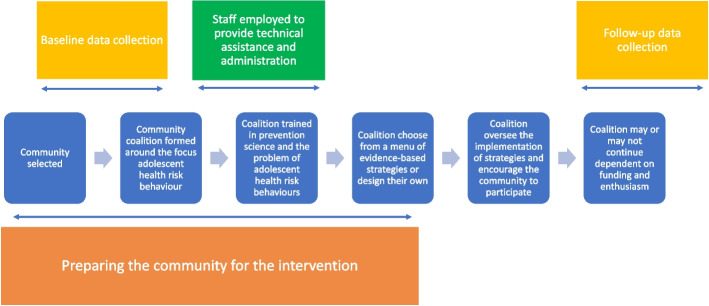
Community mobilisation intervention example Legend: A visual representation of the potential stages of implementing and evaluating a community mobilisation intervention. This is an example based on broad commonalities across interventions and is not based on one particular intervention programme

Community mobilisation efforts explicitly seek to affect community-level influences through changes in policies, practices, organisations and other features of the social or physical environment that may impact the health outcome or behaviour [20], signifying a shift away from individual behaviour change to a focus on the social determinants of health [21]. The premise being that if adolescents have a stronger affiliation with their community, and there are fewer opportunities to engage in risky behaviours and more opportunities for health promoting behaviours, then adolescent MRB within a given community will decrease [22]. These approaches may still include components which address individual behaviours (e.g. health promotion programmes within schools), but they seek to combine these with other structural factors (e.g. policy changes) as well as strong cross-community collaboration as part of a package of measures that are chosen and monitored by community stakeholders. The aim of the community mobilisation intervention is to be uniquely relevant to each community to see greater benefit for adolescent health, as the involved stakeholders understand what will work best to create changes in their community [23]. Figure 1 is a visual representation of one possible way community mobilisation interventions may be implemented and evaluated.

There is systematic review evidence suggesting that higher levels of community involvement in a public health intervention are linked to more beneficial effects and positive trends across a range of outcomes [24]. There is also some evidence to support the role of community mobilisation efforts in preventing health risk behaviours. For instance, such interventions have resulted in reductions in high-risk alcohol consumption and alcohol-related injuries [25]; alcohol-impaired driving [26]; uptake of smoking in young people [17] and youth violence [27]. Researchers have highlighted that with adequate resources and training, support from within the community and the adoption of evidence-based strategies, community mobilisation approaches show promise as an effective vehicle for addressing adolescent multiple risk behaviour [20]. Further, community-mobilisation efforts are also thought to be well suited to achieving health equity [28], due to ‘shared decision making’ [29] and the incorporation of ‘upstream’ or structural elements [30]. However, this has yet to be explored in relation to adolescent multiple risk behaviour interventions.

There are also significant challenges in implementing and evaluating such approaches, which is unsurprising given the dynamic set of social interactions and relational complexity one might expect in community-centred interventions [31]. These implementation challenges include a lack of community interest and long-term engagement, design inadequacies, inflated expectations, and weakness in planning and implementing the interventions [20, 32, 33]. Tensions and different expectations between scientists and community members as well as the practical difficulty in managing multiple components and stakeholder interests have also been cited as issues [12].

Evaluation is equally challenging [34], which is reflected in the lack of empirical evaluations of community mobilisation approaches compared to less complex (often education delivery) interventions focused at the individual level [15]. There is uncertainty around how long it might take to see an impact on behaviours, although it is expected to be a lengthy process. If changes in behaviours are found, the chain linking any effects on health risk behaviours to the coalition efforts is so long and complex that causal attributions become challenging [15]. These barriers have meant community mobilisation interventions have been missing from systematic reviews such as the aforementioned adolescent MRB review [13], as they are often evaluated through alternative methods to randomised controlled trials (RCTs) such as quasi-experimental studies. There has been one key review of the *Communities that Care* (CTC) community mobilisation process, including a narrative synthesis of its implementation globally [35]. Although not meeting the inclusion criteria for our document selection, this report proved to be useful during our broader realist review in mapping out all the iterations of CTC and providing reflections on why it was successful in some countries but not others.

## Rationale for realist review

There is a strong rationale for an alternative review approach that moves beyond effectiveness measures and speaks to the complexities and challenges surrounding the delivery and evaluation of community mobilisation efforts [36]. The realist approach seeks to make sense of ‘what works, for whom, under what circumstances.’ It does this by developing and testing hypotheses about how contexts and mechanisms interact to produce outcomes [37]. Within realist approaches, ‘context’ refers to the physical, social, economic and cultural space within which an intervention is embedded [38]. ‘Mechanisms’ are defined as ‘the real but invisible forces that make the programmes work (or not). Mechanisms are not particular components of the programme but rather, they are the 'reactions the participants have to the resources the programme offers’ ([39], page 244, [40]). Outcomes can be intended health outcomes of which the intervention is targeted, or unintended consequences of the intervention. Interventions under the realist approach are viewed as producing outcomes not directly, but as a consequence of individuals engaging with these resources in a certain context to bring about change [37]. Context can influence the extent to which these resources trigger mechanisms (e.g. because of the structure, culture or social norms) and whether that is enough to produce observable outcomes [37]. In this review, we are concerned with examining configurations of contexts and mechanisms to determine how they bring about outcomes to articulate *how* community mobilisation interventions work for adolescent health. Therefore, a realist review was chosen as the most appropriate methodological approach.

There is a pre-specified protocol for our review which has been registered (PROSPERO registration number: CRD42020205342) and published [36]. This includes detail on the research aim, realist review approach and methodological stages, search strategy, inclusion criteria and plans for dissemination. We provide an outline of the methods here and note any modifications to the original protocol. The methodological steps are described below, using the RAMESES (Realist and Meta-narrative Evidence Synthesis: Evolving Standards) [40] to guide our approach.

## Review aim

Our aim is to use a theory-driven evidence synthesis to assess how and why community mobilisation interventions work/do not work to prevent adolescent multiple risk behaviour and in what contexts. We are also interested in ‘who’ these interventions work for, so we can understand the impact of these types of interventions upon existing health inequalities. Although the focus of the review is adolescent multiple risk behaviour, we aim for our review to develop transferable learning about community mobilisation approaches more broadly in public health research and adolescent health interventions. The realist review was guided by the following sub-questions:

1. What are the mechanisms through which community mobilisation interventions produce outcomes?
2. What are the key contextual influences that determine whether the mechanisms produce outcomes?

## Methods

### Study design

**Fig. 2:**
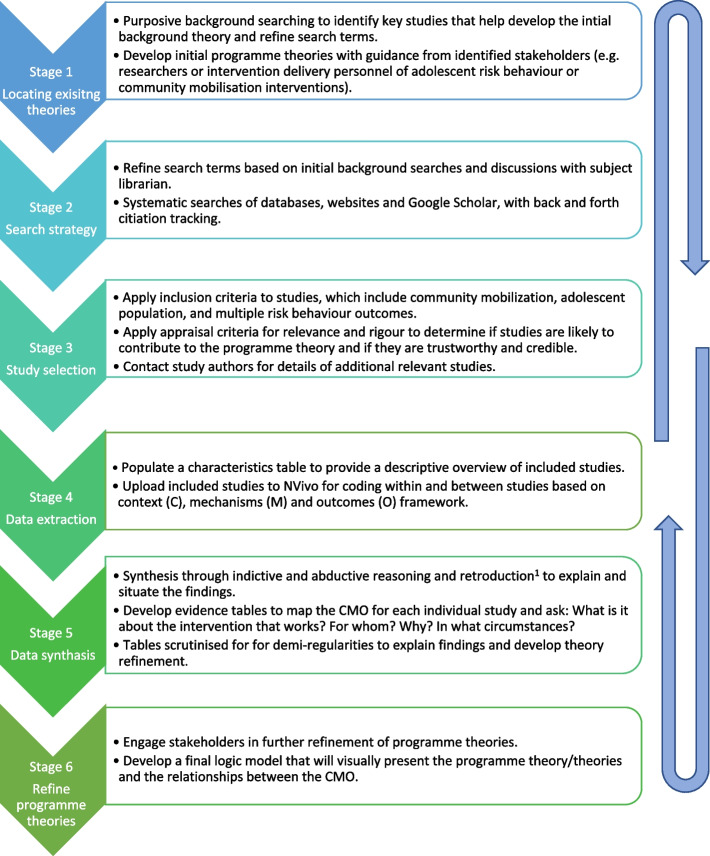
Stages of realist review Legend: Summary of stages of realist review adapted from Power et al. [ 42 ]. This depicts the steps for developing the initial programme theory, searching for evidence and synthesising the data with the input of key stakeholders (researchers and intervention delivery practitioners within the field of community mobilisation and adolescent health). Retroduction refers to inferences made through interpreting the data about the underlying causal mechanisms

This review was structured around the five review stages outlined by Pawson et al. [41] and has been informed by other realist review protocols in the field [42, 43]. Figure 2 is a diagram of the review process adapted from Power et al. [42].

## Stages of realist review

### Identifying working programme theories

We began our realist review by conducting scoping searches to identify potentially relevant documents that may help to explain how community mobilisation interventions work to address adolescent multiple risk behaviour. The search included academic databases (MEDLINE, PubMed, Web of Science), UK health websites and grey literature databases (OpenGrey, the King’s Fund, The Health Foundation), as well as Google Scholar. Broad search terms were used at this scoping stage (e.g. “community mobilisation”, “community coalition”, “youth”, “adolescence”, “health risk behaviour”, “substance use”, “antisocial behaviour”) and back and forth citation tracking was used until we developed a core set of documents to help build the initial programme theory framework [44]. This initial search was not designed to be exhaustive: this stage in the theory development is expected to be a ‘rough starting point’ that will be refined throughout the realist review process [45].

We reviewed the initial documents to develop a preliminary programme theory model (Additional file 1) and a long list of working theories that related to different elements in the model, with a description of the theories and how they explained how the intervention worked. For example, we had a theory specifically about the context of a geographical location that had social problems resulting from adolescent multiple risk behaviour and how that may trigger certain mechanisms within the intervention. We used this model and a preliminary theories document to engage in discussion with five key stakeholders (who remain anonymous) identified through our citation searching. Each stakeholder had experience or expertise in delivering community mobilisation approaches to adolescent multiple risk behaviour, with some being co-authors to the final sample of interventions and/or the preliminary set of documents. Several potential stakeholders were contacted through their organisational email address. Those that responded engaged in an online videocall with the lead reviewer to discuss the realist review. The stakeholders commented on the programme theory so far, indicated other studies that may be of use and gave insight into the experience (potential contexts and mechanisms) underlying the published work that then elicited alterations to the programme theory model. Changes were made to the programme theory model and the long list of different theories as a result of these conversations and during the data synthesis process. Additional detail on how the programme theory model evolved is in Additional file 1.

### Search strategy

At this stage, we conducted more formal searches to identify literature and evidence. We searched the following databases: PubMed; MEDLINE; PsycINFO; Web of Science; CINAHL; and Sociological Abstracts, from their inception. We also searched grey literature on OpenGrey and external expert organisations and charity websites. We searched ProQuest for unpublished theses and dissertations. We used Google Scholar for citation searching and to check the reference lists of relevant papers.

We developed search terms and syntax from the initial background search in Stage 1, discussions within the research team and previous experience with search strategies used in two multiple risk behaviour systematic reviews [11, 13]. Search terms will include MeSH terms and free text related to “community mobilisation”, “adolescence” and a range of multiple health risk behaviours. No date restrictions will be used and only studies in the English language were assessed for eligibility. The search strategy can be found in Additional file 2.

While formalised and systematic, the sampling approach in realist reviews remains purposive to answer specific questions and develop theories [41]. Therefore, the process was iterative [41], with back-and-forth citation tracking remaining a key part of the iterative search strategy [46].

### Study selection

#### Relevance and rigour assessment

Two independent reviewers (LT and CK) assessed each document to determine its relevance to our review, extracted information on a predetermined form and appraised the quality of each document. The form containing the relevant verification, data extraction and rigour appraisal can be found in Additional file 3. To be deemed *relevant* to our research aim, the study had to:

- Be written in the English language
- Describe a *community mobilisation intervention* (i.e. with a community coalition element)
- Describe an intervention targeting *two or more* adolescent health risk behaviours from a predetermined list, including substance use (tobacco, alcohol and drug use), violence and risky sexual behaviour. The behaviours did not necessarily have to be measured as outcomes for the document to be relevant, but needed to be the focus of the intervention to prevent them in young people aged 10–19 years. Adults may be included but the focus should be on adolescents.
- Include measurement of an outcome or result (qualitatively or quantitatively). Our outcome of primary interest, and what we based our programme theory on, is adolescent health risk behaviour outcomes. Other health, education or social outcomes or risk and protective factors (e.g. school connectedness, time spent with parents), community-level outcomes (e.g. changes in community crime levels) or process and implementation outcomes (e.g. the degree to which the intervention was successfully delivered or level of coalition functioning) are also of interest as they are intended to lead to reductions in population level adolescent MRB.

We included these types of outcomes because community mobilisation efforts often take many years to realise behavioural outcomes. There is a wealth of evidence on implementation outcomes relating to these interventions, which we deemed potentially useful in answering our question of *how* the interventions work in what circumstances. Some documents were not classified as relevant due to not meeting the ‘measuring outcomes’ criterion. They either described intervention challenges or outlined the implementation process, usually of interventions for which we did obtain documents presenting outcomes. We did not extract contexts, mechanisms and outcomes (CMOs) or data from these excluded documents, but some did contribute to theory development by adding contextual and mechanistic insight in a similar way to stakeholder input, which is acceptable within a realist review.

To appraise the documents’ quality (i.e. the *rigour* assessment), we used an adapted version of the Mixed Methods Appraisal Tool (MMAT) and the Critical Appraisal Skills Programme (CASP). We created a quality assessment form guided by Minian et al.’s [47] form adapted for their realist review on multiple health behaviour interventions for smoking cessation outcomes. Each study was assessed by two reviewers, with sets of questions for different study designs to derive a quality score: RCTs (eight questions), non-randomised studies (10 questions), qualitative studies (10 questions) and mixed method studies (six questions), all scored through a nominal scale (Yes/No/Not clear). An overall quality score was calculated for each study by taking the number of ‘Yes’ answers and dividing it by the number of questions. Scores were derived using the following descriptors: 0–25% (*), 26–50% (**), 51–75% (***) and 76%+ (****).

### Data extraction and synthesis

Data were extracted using a predesigned form collecting information about the study country and geographical area, the intervention activities, the coalition composition and the participants in the study. Data analysis was informed by the constructivist epistemological position, which is a paradigm aligned with the realist approach [39]. We structured our analysis around the recognition that knowledge is constructed by an individual’s perceptions. On a practical level, data analysis therefore began with reviewers coding information in the documents, coding any text that could relate to possible contexts, mechanisms and outcomes (CMOs) and including these in the data extraction form—being open to as many possibilities as we could think of. We met to discuss our long list of codes and searched for any similarities and conflicts in our initial coding.

Guided by realist review training, we then developed a diagrammatic tool using the initial codes, which we then systematically applied to each document independently. This process helped us build CMO configurations as it encouraged thinking about the causal pathways that resulted in the outcomes. In realist research, subjectively constructed knowledge is built on abductive and retroductive logic. Abduction is the creative inference required to imagine underlying causal mechanisms and retroduction is the theorising needed to develop a way of ‘testing’ whether these mechanisms exist [48]. Abduction has been described as a form of reasoning which examines evidence and makes inferences based on ‘educated guess work’ and ‘informed hunches’ about the causal factors linked to that evidence ([38], p.135). Therefore, displaying the possible contexts, mechanisms and outcomes of the interventions in a visual way allowed us to manoeuvre the different components and ‘test’ potential causal pathways. Each reviewer created their own set of CMO configuration diagrams, which were then used in multiple discussions until we agreed on the most salient set of CMOs for each document. An example of the diagram for one of the documents appears in.

Data synthesis involved assessing the documents and diagrams and compiling CMO configurations to map patterns of pathways of intervention functioning that help explicate programme theory [49]. Synthesis was driven by realist analytical approaches. For instance, coding involved *deductive* reasoning whereby we revisited our preliminary programme theory to look for alignment and conflicts within the data. We sought to be inductive in approach through grounding our reasoning in the data and being open to new and potentially challenging causal pathways. Finally, critical for realist synthesis, we practised retroduction and abduction in our synthesis, by selecting different combinations of interventions and meeting to discuss the similar or different causal pathways within each group. The CMO diagrams were used simultaneously with textual coding to explore commonalities and contrasts across the interventions and interpret potential causal mechanisms. For example, if particular mechanisms appeared to be meaningful within one intervention, this was applied to other interventions and the documents were revisited with this particular mechanism in mind. This interpretive and iterative process resulted in a set of middle range theories that contributed to the wider understanding of how the interventions work (or do not work), with evidence presented from the relevant studies.

## Findings

### Document characteristics

**Fig. 3:**
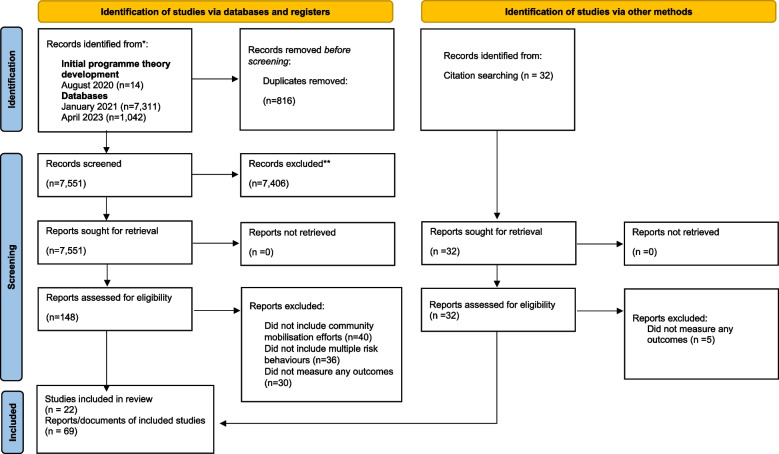
PRISMA flow diagram of included studies Legend: Flow diagram showing the stages of searching, screening and inclusion that resulted in our final sample to review

A total of 69 documents describing 22 different interventions were obtained for this review. Figure 3 shows the PRISMA flow diagram of how we reached that sample size. Where single interventions were described by multiple documents, the study team selected a core set of documents for data extraction, which resulted in a final focused sample of 35 documents. Decisions about these documents were made based on relevance to our research question and whether they were felt to add new insights about the intervention. Many documents repeated outcome measures at different time points and therefore added little extra detail to our analysis. We familiarised ourselves with the documents not selected for data extraction, being open to potential key contextual or mechanistic data; although data were not directly extracted from them, the information contained within them still informed our interpretations. Additional file 4 contains a table reporting the final sample of interventions, by the number of documents related to them and the type of outcome they report (e.g. implementation outcomes, community-level outcomes or individual adolescent health risk behaviour outcomes).

Table 1 presents the general characteristics of the sample documents selected for data extraction.

**Table 1:** Table of sample characteristics

**Study characteristic**	***N* (%)**
*Year of first publication associated with the intervention*	2020 onwards (*n* = 3)
	2010–2019 (*n* = 5)
	2000–2009 (*n* = 10)
	1990–1999 (*n* = 4)
*Country of intervention*	United States (*n* = 13)
	United Kingdom (*n* = 2)
	Thailand (*n* = 1)
	Iceland (*n* = 1)
	Lithuania (*n* = 1)
	Chile (*n* = 1)
	Netherlands (*n* = 1)
	Mexico (*n* = 1)
	Australia (*n* = 1)
*Quality assessment score*	^****^ (*n* = 2)
	^***^ (*n* = 15)
	^**^ (*n* = 5)
	^*^ (*n* = 0)
*Study Design*	Randomised controlled trial (*n* = 4)
	Quasi-experimental (*n* = 3)
	Observational study (*n* = 7)
	Evaluation case study (*n* = 3)
	Process evaluation (*n* = 4)
*Number of behaviours targeted*	Two behaviours (*n* = 2)
	Three behaviours (*n* = 8)
	Four behaviours (*n* = 7)
	Five behaviours (*n* = 5)
*Outcomes*	Health risk behaviour outcomes (*n* = 14)
	Implementation, process or coalition functioning outcomes (*n* = 8)

The table contains broad study characteristics across the sample

Table 2 contains the relevant extracted data, example CMO configurations and quality assessment scores for each of the 22 interventions under review.

**Table 2:** Table of characteristics and extracted data

Study and location, *Intervention name*	Intervention content and results	Contexts identified	Mechanisms identified	Key CMO configurations	Rigour/Quality assessment
Kristjansson et al. 2020 [22, 50] (Iceland) *Icelandic Prevention Model (IPM)*	**Description** In response to the high rates of adolescent substance use in Iceland, policymakers and social scientists came together to explore bottom-up collaborative approaches to substance use prevention and developed the Icelandic Prevention Model (IPM). Grounded in theories of social deviance, rather than behaviour change models, IPM was based on the theory that most individuals are capable of deviant acts, but only under certain environmental and social circumstances will those acts become common patterns among dominant groups of adolescents. The goal of the approach from the outset was to mobilise society as a whole in the struggle against adolescent substance misuse, with emphasis on community engagement and collaboration leading to long-standing and gradual environmental and social change rather than short-term solutions. **Coalition information** **Results** The evaluation demonstrated a significant difference in group trends over time in smoking and alcohol use, parental monitoring, party lifestyle, and participation in organised sports, with the treatment group being favoured in all instances. Since the original development of the model, Iceland has led the decline in substance use in all of Europe. In 2015, the rate of ever smoking tobacco was 46% among 10th grade adolescents in Europe but had plunged to 16% in Iceland; average rates of current alcohol use were 48% in Europe but 9% in Iceland; and average rates of lifetime use of cannabis substances remained at 16% in Europe, similar to 1999, but declined to 5% in Iceland. Iceland had also witnessed large reductions in risk factors and strengthening of protective factors. For example, 10th grade students reporting parents knowing with whom they spend time in the evenings increased from ~ 50% in 2000 to just over 74% in 2016.	1. A model of ‘guiding principles’ that are flexible and not prescriptive in terms of intervention choice, but guide communities through a framework they can continually come back to. 2. A diverse coalition of policymakers, experts and local people. 3. Commitment to data collection and establishing community readiness 4. Weaving the guiding principles throughout different systems (e.g. schools, family, policy)—multi-component approach 5. High adolescent substance use 6. Developing a whole-country approach (relatively small country and an island) 7. Focus on social theories and risk and protective factors over and above risk behaviour change	• Community empowerment • Credibility—through strong consideration of local data (e.g. annual surveys) • Flexibility and relevance to each community • Collaboration and strengthening connections between family, school and community (establishing cohesion and unified focus) • Intervention champions (mechanism resource)	1. Model of guiding principles integrated into all fractions of adolescent life (context) ➔ **empowered** community members and local stakeholders to make their own decisions that align with the principles (mechanism) ➔ resulting in successful implementation outcomes and then improvements in protective factors and reductions in adolescent substance use (outcomes). 2. Model of guiding principles integrated into all fractions of adolescent life (context) ➔ encourage strong **collaboration** across schools, families, policy and practice, all ‘on the same page’, bolstered by the mechanistic resource or intervention champions who worked to maintain collaboration ➔ resulting in successful implementation outcomes and then improvements in protective factors and reductions in adolescent substance use (outcomes). 3. Commitment to establishing community readiness ➔ using relevant and timely data through surveys (resource) which enhance the credibility of the approach (mechanism reaction) and ensured the community would respond well to the intervention ➔ resulting in successful implementation outcomes and then improvements in protective factors and reductions in adolescent substance use (outcomes). 4. A diverse coalition of policy and prevention stakeholders and community members ➔ balances expertise and knowledge with community relevance and empowerment—ability to enact change and collaborate ➔ resulting in successful implementation outcomes and then improvements in protective factors and reductions in adolescent substance use (outcomes).	^***^
Libuy et al. 2023 [51] (Chile) Icelandic Prevention Model delivered in Chile	**Description** In 2018, six municipalities in Greater Santiago, Chile, implemented the Icelandic prevention model, including structured assessments of prevalence and risk factors of substance use in 10th grade high school students every 2 years. Totally, 7538 participants were surveyed in 2018 and 5528 in 2020, nested in 125 schools from the six municipalities. The survey underwent a process of cultural and linguistic adaptation involving members of the municipal prevention teams, experts, and piloting among adolescents to ensure understanding, adequacy, and representation of relevant substances commonly used by adolescents in Chile **Coalition information** No information about the coalition(s), but if implementing the IPM, coalitions should be a mixture of community members and stakeholders with policy and prevention expertise. **Results** Lifetime alcohol use decreased from, past-month alcohol use decreased from, and lifetime cannabis use decrease from. Several risk factors improved between 2018 and 2020: staying out of home after 10 p.m., alcohol use in friends, drunkenness in friends, and cannabis use in friends. However, other factors deteriorated in 2020: perceived parenting, depression and anxiety symptoms, and low parental rejection of alcohol use. The decrease of substance use prevalence in adolescents was attributable at least in part to a reduction of alcohol use in friends. This could be related to social distancing policies, curfews, and homeschooling during the pandemic in Chile that implied less physical interactions between adolescents. The increase of depression and anxiety symptoms may also be related to the COVID-19 pandemic. The factors rather attributable to the prevention intervention did not show substantial changes (i.e. sports activities, parenting, and extracurricular activities). In conclusion, the study showed a marked reduction of alcohol and cannabis use in adolescents in six municipalities of the Greater Metropolitan Region of Santiago in Chile, which have started to implement the Icelandic prevention model since 2018. However, these results can be influenced by measures to control the pandemic, and are not necessarily attributable to the effectiveness of the model. The implementation of the prevention model is an ongoing process, that in the future may need the transfer of further components.	1. A model of ‘guiding principles’ that are flexible and not prescriptive in terms of intervention choice, but guide communities through a framework they can continually come back to. 2. Commitment to data collection, establishing community readiness and transferability of the intervention to Latin America. 3. Focus on social theories and risk and protective factors over and above risk behaviour change	• Community empowerment • Credibility—through strong consideration of local data (e.g. annual surveys) • Flexibility and relevance to each community • Collaboration and strengthening connections between family, school and community (establishing cohesion and unified focus)	1. Model of guiding principles integrated into all fractions of adolescent life (context) ➔ **empowered** community members and local stakeholders to make their own decisions (relevant to the Latin America context) that align with the principles (mechanism) ➔ resulting in successful implementation outcomes and then improvements in protective factors and reductions in adolescent substance use (outcomes). 2. Commitment to establishing community readiness and adapting the intervention to Latin America ➔ using relevant and timely data through surveys and collaborating with Iceland colleagues (resource) which enhance the credibility of the approach (mechanism reaction) and ensured the community would respond well to the intervention ➔ resulting in successful implementation outcomes and then improvements in protective factors and reductions in adolescent substance use (outcomes). 3. Implementation alongside Covid-19 pandemic measures ➔ supported some mechanisms (collaborations across policy and schools) and made others a challenge (e.g. extracurricular activities) ➔ A challenge to determine whether it was pandemic measures or the intervention that impacted on risk and protective factors and reductions in substance use.	^***^
Asgeirsdottir et al. 2021 [52] (Lithuania) Icelandic Prevention Model in Lithuania	**Description** With the aim of improving the social conditions of young people in Lithuania and to decrease substance use related harms, in 2006 the Lithuanian cities Kaunas, Klaipeda and Vilnius begun implementing the IPM via their participation in the Youth in Europe project in cooperation with Icelandic Centre for Social Research and Analysis (ICSRA) and the Stockholm-based European Cities against Drugs Organization.7–9 The implementation of the IPM was aligned with the IPM in Iceland, including the key pillars of the model.1,4,5 In all the cities, the work has been evidence-based, surveys have been carried out on a regular basis and data collected and processed using ICSRA standards. Survey findings have been disseminated throughout communities and schools and results presented to parents and other local stakeholders. Municipal administration and community stakeholders have set goals, and formed policy and practice based on the study findings. Major focus areas include emphasis on increasing parental monitoring, parental involvement, youth participation in organised and supervised leisure activities, and preventing unsupervised parties and late outside hours among young people. Data collected from repeated, comparative cross-sectional self-report surveys conducted among a total of 30 572 10th graders in the cities of Kaunas, Klaipeda and Vilnius, Lithuania, from 2006 to 2019, were analysed. Cochran– Armitage test for linear trend and analysis of variance for linear trend was used to assess time-trends in prevalence of substance use and mean levels of primary prevention variables over time. **Coalition information** No information about the coalition. **Results** Following the implementation of IPM rates of cigarette smoking and the use of alcohol, cannabis and amphetamine has decreased among 10th graders in Lithuania’s three largest cities and simultaneously preventive variables targeted have improved. Similar to Iceland, primary prevention variables were related to substance use in the expected direction, with the exception of organised sports participation, which was not associated with less likelihood of alcohol, cannabis and amphetamine use	1. A model of ‘guiding principles’ that are flexible and not prescriptive in terms of intervention choice, but guide communities through a framework they can continually come back to. 2. Commitment to data collection, establishing community readiness and transferability of the intervention to Latin America.	• Community empowerment • Credibility—through strong consideration of local data (e.g. annual surveys)	1. Commitment to model of guiding principles integrated into all fractions of adolescent life (context) ➔ empowered community members and local stakeholders to make their own decisions (relevant to the Baltic country context) that align with the principles (mechanism) ➔ resulting in successful implementation outcomes and then improvements in protective factors and reductions in adolescent substance use (outcomes). 2. Commitment to establishing community readiness and adapting the intervention to Lithuania ➔ using relevant and timely data through surveys and collaborating with Iceland colleagues (resource) which enhance the credibility of the approach (mechanism reaction) and ensured the community would respond well to the intervention ➔ resulting in successful implementation outcomes and then improvements in protective factors and reductions in adolescent substance use (outcomes).	^***^
Shrestha 2019 (USA) [53] *Community Prevention and Wellness Initiative (CPWI)*	**Description** The primary long-term outcome of interest for CPWI is reducing youth behavioural problems, especially underage drinking among 8th and 10th graders. Five step process similar to SPF. Once CPWI communities are selected, they take part in a five-step strategic planning process that uses survey and archival data to help community coalitions coordinate, assess, plan, implement, and evaluate youth substance use prevention services. CPWI communities are expected to conduct programmes in a culturally competent manner, build community capacity for prevention programming, and maintain a long-term vision for sustainability during planning and implementation efforts. **Coalition information** • Pre-existing coalitions and new coalitions (but funding is specifically for youth problems) • 6 coalitions across 18 communities, with between 8–12 individuals on each • Mix of strategic leaders, community residents and young people **Results** CPWI was effective in reducing adolescent alcohol use and some family and community risk factors. Higher rates of cigarette use in CPWI communities and mixed results for marijuana and combined use.	1. Relaxed social norms towards marijuana use 2. Coalitions form (or are pre-existing) then apply for funds specifically for adolescent health risk behaviour 3. Established funding structure and public health approach on a national level	• Technical assistance • Prevention training • Motivation for intervention delivery • Single focus (clarity) • Organisation	1. Relaxed social norms towards some adolescent health risk behaviours (e.g. through marijuana legalisation) (context) ➔ motivation to engage with community strategies limited (mech) ➔ difficulty in reducing health risk behaviours 2. Coalition is specifically formed for the purpose of rallying around the problem of high rates of adolescent health risk behaviours (context) ➔ the coalition will have a single focused aim, which leads to greater organisation and empowerment through clarity (mech) ➔ positive implementation/health risk behaviour outcomes (outcome) 3. Established funding structure, time spent on community readiness and a history of adolescent public health (context) ➔ technical assistance and prevention training will be provided to trigger motivation for the intervention delivery (mech) ➔ resulting in positive outcomes (outcome)	^****^
Galai et al. 2018 [54] (Thailand) *Connect to Protect (C2P) Thailand*	**Description** This approach works to mobilise the community, through the formation of coalitions, composed of local leaders from various sectors, that develop and implement structural changes at the community level to address key health issues: illicit drug use (methamphetamine) and HIV risk behaviours (risky sex and drug use), alcohol use, cannabis use, selling MA in 14–24-year-olds. **Coalition information** • A local community coalition was assembled in each of the intervention districts. • Representatives from the local communities, public health and education sectors, police officials and religious leaders. **Results** Reduction in methamphetamine use in both intervention and control with no meaningful difference. Shift in approach from legal to public health framework. Activities were triggered in both intervention and control communities.	1. Community problems with adolescent MRB 2. Societal context (i.e. developing country, ‘war on drugs’ legal framework) 3. Coalition newly formed of local ‘leaders’ 4. Lack of public health delivery system 5. Rural town 6. Limited funding	• Motivation through shame and fear • Low understanding of prevention science • Developing and designing own approaches	1. Community problems ➔ motivation ➔ implementation outcomes and unintended consequences 2. Societal context/legal framework and public health delivery system ➔ low understanding of prevention science ➔ implementation outcomes and ineffective programmes for adolescent MRB 3. Limited funding ➔ difficulty developing and designing programmes among the coalition ➔ low motivation and organisation ➔ ineffective against adolescent MRB outcomes 4. Coalition members community leaders in a newly formed coalition ➔	^***^
Hallfours et al. 2002 (USA) [23] *Fighting Back, from The Robert Wood Johnson Foundation*	**Description** Fighting Back, initiated by RWJF promoted the notion of comprehensive approach adolescent health risk behaviours. The aim was that local political, business, and grass-roots leaders all come together around a community “table” to assess the substance abuse problems in their community and to develop a comprehensive, coordinated response. Although communities were encouraged to devise their own programmes based on local context and needs, they were required to develop a single, community-wide system of prevention and treatment, which at minimum included: (1) public awareness; (2) prevention, targeted especially at youth and children; (3) early identification and intervention; and (4) treatment and relapse prevention **Coalition composition** Newly formed coalition with community representatives and strategic leaders. **Results** Strategies aimed at either youth or community/ prevention outcomes showed no effects, while strategies to improve adult-focused outcomes showed significant negative effects over time, compared to matched controls. Coalitions with a more comprehensive array of strategies did not show any superior benefits, and increasing the number of high-dose strategies showed a significant negative effect on overall outcomes.	1. National framework supporting community mobilisation 2. Funding structure for coalition-based prevention activities and technical assistance 3. Grassroots coalitions applying for funds (community representatives/strategic leaders on coalition)	• Flexibility over own strategies • Ownership and commitment to intervention • Empowerment through designing own strategies • Motivation to continue with intervention	1. Coalition starts with community members applying for funds in a grassroots way (context) ➔ design and develop their own chosen strategies relevant to the community problem of adolescent health risk behaviours, leading to a sense of ownership, empowerment, flexibility and commitment to intervention ➔ positive community level and health risk behaviour outcomes **RIVAL THEORY 2.1:** Coalition starts with community members applying for funds in a grassroots way (context) ➔ design and develop their own chosen strategies relevant to the community problem of adolescent health risk behaviours ➔ lack of structure, oversight and knowledge of evidence based programmes ➔ implementation of ineffective programmes and no change in youth outcomes 2. Funding available to be applied for by coalitions ➔ technical assistance and the delivery of a wide range of strategies, leading to stronger motivation for the intervention as coalition and community members know there is sustainability in their efforts ➔ leading to positive community level and implementation outcomes.	^***^
Brown 2015 [55] and Feinberg 2010 [56] and Chilenski 2019 [57] and Feinberg 2004 [58] (USA) *Communities that Care (CTC) Pennsylvania*	**Description** The CTC strategy is an intervention approach focused on adolescents aged 10–14 years, providing guidance through training and technical assistance to (1) organise members of communities into collaborative coalitions, (2) develop coalition capacities such as the knowledge and skills to use a science-based approach to prevention, (3) conduct an epidemiological assessment of adolescent risk and protective factors to identify community needs, (4) prioritise community needs and select evidence-based programmes (EBPs) to address those priorities, and (5) implement EBPs and monitor community prevention efforts and outcomes to ensure a high-quality implementation and the achievement of goals. The majority of the programmes are school-based. Since 1994, the Pennsylvania Commission for Crime and Delinquency (PCCD) has funded the development and training of over 125 CTC coalitions. Supporting the success of CTC coalitions are coalition mobilisers and technical assistance providers. Coalitions hire a mobiliser to help organise day-to-day operations. **Coalition composition** 127 coalitions—mixed sizes and compositions although the aim is to include a mix of strategic partners and community residents. There is an oversight body the coalitions report to. **Results** Community coalitions can affect adolescent risk and protective behaviours at a population level when evidence-based programmes are utilised. CTC represents an effective model for disseminating such programmes. Analyses revealed that CTC school districts had significantly lower levels of adolescent substance use, delinquency, and depression. This effect was small to moderate, depending on the particular outcome studied. Overall effects became stronger after accounting for use of evidence-based programmes; there are likely differences in implementation quality and other factors that contribute to the observed overall small effect size. Future research needs to unpack these factors.	1. National framework supporting community mobilisation 2. Funding structure for coalition-based prevention activities and technical assistance 3. Step by step process to follow to mobilise community and deliver strategies 4. Small (often rural) towns with distinct ‘community’ boarders	• Menu of evidence based programmes • Motivation to continue (funding provided and previous success) • Trust and credibility of programmes menu • Low flexibility (to design own strategies)	1. National funding structure (context) ➔ coalition can pay for technical assistance and the delivery of a wide range of strategies (resources) ➔ stronger motivation for the intervention as coalition and community members know there is sustainability in their efforts (mech reasoning) ➔ positive community level and implementation outcomes. 2. There is a long history and established method for adolescent health coalitions (context) ➔ a menu of strategies and school-based programmes (mech resource) ➔ promotes trust among coalition and community members (reasoning) ➔ leading to successful implementation and reductions in adolescent health risk behaviours. 3. There is a long history and established method for adolescent health coalitions (context) ➔ a menu of strategies and school-based programmes (mech resource) ➔ limits flexibility and empowerment of the coalition members (reasoning) ➔ the focus is largely on the evidence based programmes to deliver successful outcomes, not the community members. Whatever they choose will hopefully lead to successful outcomes 4. Small (often rural) towns with distinct ‘community’ boarders ➔ clarity and focus on schools in a defined area ➔ strong sense of ‘community’ and the needs ➔ easier to determine outcomes and assign them to intervention	^****^
Fagan 2009 [59] and Oesterle-2015 [60] and Oesterle-2018 [61] and Hawkins 2012 (USA) [62] *Communities that Care (CYDS)*	**Description** Communities That Care (CTC) is a system for guiding communities to choose, install, and monitor tested and effective preventive interventions to address elevated risks and suppressed protective factors affecting youth aged 10–14 years. The CTC system is expected to produce community-wide changes in prevention system functioning, including increased adoption of a science-based approach to prevention and increased use of tested, effective preventive interventions that address risk and protective factors prioritised by the community. The majority of the programmes delivered are school-based. Coalitions chose to deliver 18 school based programmes implemented across 12 communities. **Coalition composition** 516 members over 12 coalitions, which includes strategic partners and community residents. 24 small towns in 7 states, matched within state. **Results** Several implementation and health risk behaviour outcomes over many years, most seeing a reducing in risk behaviours. For example, the results indicated that intervention communities enacted, on average, 90% of the core components of the CTC system, and achieved high rates of implementation fidelity when replicating school, afterschool, and parent training programmes [59]. In 2012, mean levels of targeted risks increased less rapidly between grades 5 and 10 in CTC than in control com- munities and were significantly lower in CTC than control communities in grade 10. The incidence of delinquent behaviour, alcohol use, and cigarette use and the prevalence of current cigarette use and past-year delinquent and violent behaviour were significantly lower in CTC than in control communities in grade 10 [62]. In 2015, CTC had an overall effect across lifetime measures of the primary outcomes for males, but not for females or the full sample, although lifetime abstinence from delinquency in the full sample was significantly higher in CTC communities (ARR = 1.16) [60]. Finally, in 2018, The CTC system increased the likelihood of sustained abstinence from gateway drug use by 49% and antisocial behaviour by 18%, and reduced lifetime incidence of violence by 11% through age 21 years. In male participants, the CTC system also increased the likelihood of sustained abstinence from tobacco use by 30% and marijuana use by 24%, and reduced lifetime incidence of inhalant use by 18%. No intervention effects were found on past-year prevalence of these behaviours (Oesterle et al. 2018).	1. National framework supporting community mobilisation 2. Funding structure for coalition-based prevention activities and technical assistance 3. Step by step process to follow to mobilise community and deliver strategies 4. Small (often rural) towns with distinct ‘community’ boarders	• Community champions of the intervention • Collaboration with schools • Menu of evidenced based programmes to select • Credibility of programmes and wider intervention • Ownership of coalition members • Low flexibility (over developing own programmes) • Motivation and empowerment	1. National history and funding structure that supports community mobilisation ➔ community members (champions) act as catalysts for the intervention (mechanism) and there are leaders to motivate the community and maintain focus throughout schools upon adolescent health behaviours (mechanism reasoning) ➔ successful implementation and health risk behaviour outcomes. 2. If there is funding available at a government or local level for technical assistance and support (context) ➔ this fosters credibility and early support for the intervention (reasoning) ➔ leading to positive health behaviour outcomes 3. Funding structure to support community mobilisation (context) ➔ ensures training available for community members (resource) ➔ which maintains ownership over the project and empowers people in the community (reasoning) ➔ leading to successful implementation 4. Having a menu of strategies (mech resource) ➔ within the context of key community stakeholders and residents on the coalition (context) ➔ promotes empowerment as the coalition members, who may have little public health experience, can feel confident in choosing from a menu instead of designing their own programmes (reasoning) ➔ leading to positive health behaviour outcomes 5. The structure and methodology of a programme like CTC (mech resource) ➔ within the supportive societal context of adolescent health prevention (context) ➔ promotes credibility, organisation and motivation (mech reasoning) ➔ for quicker success in implementation and health behaviour outcomes (outcomes)	^***^
Toumbourou et al. 2019 [63] (Australia) *Communities that Care Australia*	**Description** The Australian license to implement the CTC process was purchased from the U.S. developer in 1999 and a company was formed to offer the technical assistance and resources to Australian communities. The Australian implementation of CTC was initiated using the training manuals and technical resources available in 2002, with the developer providing training updates in later years. The evidence-based programmes available for CTC sites in Australia are regularly updated by the Australian Research Alliance for Children and Youth, Prevention Science Network and listed on a searchable website (http://whatworksforkids.org.au). As relevant to adolescents, the initially developed action plans in all four communities have focused on the prevention of adolescent alcohol use (and in three communities other drug use was also targeted). The present evaluation focused on the first four Australian coalitions that completed all five phases of the CTC process. The first three communities initiated activities from 2001 to 2002. ‘Champions’ from these three municipalities approached CTC following national publicity about the process. All three communities were able to successfully raise state-government funding to complete the process. An independent process evaluation reported these communities successfully completed all five phases of the CTC process in one or more locations within their community (Kellock 2007). After completing their first five-phase cycle (in 2007), CTC Mornington Peninsula were supported by their local government to complete a further two cycles of the process. **Coalition composition** Usually a combination of community residents and strategic partners and may have civil servants They work with youth advisory boards. Individuals approach CTC and formed coalitions that then gained state-government funding. Coalitions report to an oversight Board. **Results** Implementation of the CTC process in Australia was associated with more rapid community reductions in adolescent health behaviour problems. Supporting community coalitions to adopt evidence- based interventions appears a feasible means for health psychologists to improve the health of large adolescent populations and prevent related chronic health problems in later life. Thus, an important implication arising from the current report is the feasibility of using community coalition models to enhance social capital as a means of contributing to public health.	1. High rates of adolescent health risk behaviours 2. Strong focus on adolescent health in Australia—national campaign about the intervention 3. New and established coalitions 4. CTC process interwoven within local authority and national public health strategies and personnel 5. Time spent adapting the USA programme to Australian context	• Community, local authority and strategic champions • Motivation through collaboration • Menu of evidence based programmes	1. High rates of health risk behaviours and history of adolescent health focus in Australia (context) ➔ champions working within local authorities, or community members on the board (mech resource) to spread the word about CTC and prevention strategies ➔ positive implementation outcomes and reductions in risk behaviours (outcomes). 2. A mix of new and established coalitions were formed using the CTC framework that were interwoven within local authority and national public health strategies and personnel (context) ➔ motivation and collaboration with established colleagues, schools and community members to take on board CTC ➔ positive implementation outcomes and reductions in risk behaviours (outcomes). 3. Time spent adapting USA programme to Australian context and making local authorities and communities aware of the intervention ➔ trust and credibility balanced with tailored resources ➔ positive implementation outcomes and risk behaviour outcomes.	^***^
Jonkman et al. 2005 [64], 2009 [65] and Steketee et al. 2013 [66] (Netherlands) *Communities that Care Netherlands*	**Description** Adaptation of CTC to Netherlands. The Dutch data were collected from 10 cities which implemented CTC during 2000–2006. With a grant from the Dutch government, 3 of the 10 cities started in 2000 with the implementation of the prevention system. Since 2004, their prevention work has been financed by their municipalities. The other seven communities have initiated implementation of CTC at different times between 2004–2006. The roll of the Dutch national state in implementing CtC was substantial, which is in line with the historical tradition of Dutch social policy. It was the state through the Ministries mentioned above that took the initiative of limited trial implementation. CTC staff were trained and certified through an agreement with the US distribution company of CTC. Communities That Care staff delivered training to community boards and provided technical assistance, mostly by direct contact and general meetings. Each CTC community in the Netherlands has a full-time local coordinator. Communities in the Netherlands did not receive annual financial support for programme implementation. Before the programme could be implemented the American student survey used by CTC USA had to be translated and adapted (as little as possible) to the Dutch situation. Some adaptations had to be made based on cultural differences between the United States and the Netherlands. For example we considered that according to Dutch youth culture there were too many questions on drug use and weapons and too little on protective factors. The instrument was tested and piloted for relevance and comprehension before using it as a research tool. **Coalition composition** 47 coalition members across the 5 CTC coalitions. Service professionals with CTC as part of their job roles. The aim to involve community members and young people. 10 neighbourhoods (5 intervention and 5 control neighbourhoods) located in 5 cities in the provinces. **Results** Implementation and Board functioning outcomes (evaluation outcomes contained in a Dutch document). CTC communities appear to be working in a more targeted fashion to address risk and protection, increased demand for tested programmes, creation of infrastructure to support the national databank of programmes. There was a greater degree of challenge or barriers to implementation in the NL than in the US.	1. No history or structure of intervention approaches to adolescent health risk behaviours 2. Limited history of community mobilisation and coalition approaches 3. Civil servants as sole coalition members	• Limited menu of tested strategies to select from • Low motivation and trust in strategies • Low collaboration • Split focus between job role and CTC	1. If the societal context is such that there is not a history of intervention approaches towards adolescent MRB (context) ➔ the menu of tested strategies will not be strong enough (resource) ➔ and coalition members will not be motivated by or trust alternative strategies tested in other settings (reasoning) ➔ slow collaboration and poor implementation outcomes 2. Community coalition made up largely of civil servants as a voluntary part of their role (context) ➔ split focus on other problems and not a strong enough focus on the embedded community problem of adolescent health risk behaviours and the health promotion approach will not be fired to a great enough extent (resource) ➔ low levels of organisation and motivation among the coalition ➔ poor implementation outcomes	^**^
Crow, France and Hacking (UK) [67] *Communities that Care UK*	**Description** Adaptation of the USA CTC intervention for the UK context, intended for secondary school youth aged 12–16 years. Communities That Care (CTC) projects seek to improve community security by reducing the risk of young people becoming involved in problem behaviours. The eventual aim of CTC is to reduce four problems among young people: failure at school, school-age pregnancy, drug abuse, and youth crime. Thus, the success of CTC is to be judged by reference to these four measures. However, CTC is a long-term programme that affects children as they grow up, and it may therefore be 10 years or more before its full impact can be evaluated. Each area allocated £150,000 to be used to employ a co-ordinator, training and technical support. The surveys were given to all the children attending schools that covered the CTC areas. **Coalition composition** Unclear size and details of the coalition. Intended to be a mix of community members and strategic partners. 3 areas in the pilot each with a coalition. **Results** The evaluation revealed mixed success in delivering CTC in UK. For example, there was a lack of partnership working in Northside site which made it difficult to move forward; tense history between local people and professional workers; Westside had a history of community partnership - moved forward. CTC UK needs to recognise the diverse starting positions and develop different implementation models for different types of communities. Clearly, as the evidence from this evaluation has shown, ‘no one model fits all’ and a range of different models that can be used in different situations will create opportunities to build on communities’ strengths while recognising their weaknesses. Getting and maintaining a core group of local people was achieved as a result of involving these people from the very beginning, either prior to the programme being funded, or at the Community Orientation meetings. Spending more time in the early stages to search out and identify groups and individuals therefore had positive benefits.	1. No history or structure of community-led intervention approaches to adolescent health risk behaviours 2. Lack of understanding or processes to establish community readiness 3. Civil servant staff solely on coalitions 4. Limited funds and intervention delivery structure 5. Historical mistrust between local authorities and local community 6. Lack of distinct communities	• Low motivation • Low empowerment and community grounding • Understanding of prevention science • Split focus • Low community identification	1. No history of adolescent health prevention work or established interventions (such as CTC in USA) (context) and low community readiness ➔ low motivation and acceptance of the strategies within the community ➔ implementation outcomes are not met. 2. Lack of diversity on the coalition, with mostly civil servant staff and few community members (context) ➔ a good understanding of prevention science to compliment technical assistance (mech resource) ➔ but collaboration and empowerment will not be triggered under these circumstances (mech reasoning) ➔ strategies not grounded in the local context or accepted by the community ➔ poor implementation outcomes. 3. Lack of funding for the pilot intervention to continue after a short time (context) ➔ technical assistance and prevention science training cannot continue (resource) ➔ low motivation from community coalition as they are aware that there is no sustainability in the efforts (reasoning) ➔ poor implementation outcomes. 4. If there are infrastructure issues and a lack of history of adolescent health risk behaviour interventions (context) ➔ motivation (mech reasoning) ➔ may not be triggered (or take a long time) for implementing an adapted intervention like CTC (resource) ➔ leading to poor or slow implementation success.	^**^
Bannister and Dillane 2005 [68] (UK) *Communities that Care Scottish Pilot*	**Description** Adaptation of CTC to UK context. CTC is a long-term early intervention programme that aims to ameliorate the risk factors and enhance the protective factors that international research evidence has shown to influence the likelihood that a young person will: experience school failure, school-age pregnancy, or sexually transmitted diseases; engage in drug abuse; or become involved in violence and crime. Guided by a co-ordinator and various training exercises, CTC programmes are community-led. CTC places local residents and representatives (service managers and agents) of the statutory and voluntary bodies engaged in the prevention and management of the problem behaviours exhibited by young people at the heart of its decision-making structures. **Coalition composition** Unclear coalition composition. Primarily civil servants. **Results** CTC not successfully transferred to Scottish context. Challenges and hypothesised reasons included: lack of commitment because of concerns of lack of funding at the end of the pilot, opinions that CTC programmes should be integrated into broader policy contexts such as SIPS, out-of-date programmes and process, mismatch between governance and geographical areas, length of time to collect data.	1. If the national context is such that there is no history of adolescent health prevention work or established interventions (such as CTC in USA) 2. No funding post pilot 3. Civil servants on coalition 4. Communities chosen not reflective of local authority jurisdiction	• Lack of technical assistance and prevention science training due to low funds (resource) • Low motivation from community coalition and local authority members • No sustainability of efforts • Split focus between CTC and other community issues • Limited menu of programmes • Time to collect data	1. No history of adolescent health prevention work or established interventions (context) ➔ split focus with other local community issues, lack of trust in the intervention and low commitment and motivation from key leaders and community members ➔ slow progress and poor implementation outcomes. 2. Civil servants as community coalition members ➔ empowerment and ownership not triggers ➔ poor implementation outcomes 3. No funding post pilot (context) ➔ low motivation from community coalition and local authority members as they are aware that there is no sustainability in the efforts ➔ poor implementation outcomes. 4. Communities not coinciding with local authority jurisdiction ➔ Confusion and lack of community identification ➔ poor implementation outcomes	^**^
Manger 1992 [69], Harachi 1996 (USA) [70] *TOGETHER! Communities for Drug Free Youth* (Adopting the Communities that Care strategy)	**Description** In May 1988, the authors initiated a risk-focused community mobilisation project for drug abuse prevention. That project, *TOGETHER! Communities for Drug Free Youth*, is a state-wide collaboration initially involving 28 Washington communities. The project uses current research on risk and protective factors for adolescent drug abuse as its foundation. Through training and technical assistance, communities have been mobilised to design and implement comprehensive, risk-focused plans for adolescent drug abuse prevention. *TOGETHER!* adopted the Communities That Care community mobilisation strategy for risk-focused prevention of adolescent substance abuse. **Coalition composition** 244 individuals across 36 coalitions for 36 communities, 31 remaining active after 1996. Participants represented a diverse cross-section of the communities. Most teams had a least one member from each of the following: education/early childhood education, parents, local business, local government, service organisations, existing coalitions, and law enforcement/crime prevention. Less frequently, teams had participants representing the following: recreational organisations, youth juvenile justice, the media, drug treatment providers, religious groups, and specific ethnic minority groups. **Results** Process results were reported. The activities that teams had implemented over the first year were assessed to evaluate the extent to which they were either risk focused or used the social development strategy. Three teams had not implemented any activities. The remaining teams had implemented the following activities (a team may be included more than once): • Implemented or increased support to parenting programmes for drug abuse prevention (7 teams) • Lobbied for a variety of school-based programmes (11 teams) • Advocated or lobbied for policy change (5 teams) • Conducted a community forum or workshop on prevention (5 • teams) • Sponsored a drug-free, dance for community youth (5 teams) • Conducted a one time or annual drug-free, community event (7 teams) *TOGETHER!* successfully mobilised a number of community teams to engage in prevention planning and action using a risk-reduction approach. The process evaluation data suggest that higher implementation rates may be produced if community planning teams are provided with greater direction and training with regard to accountability and organisational development issues. Technical assistance to teams should take into account turnover in team membership and provide teams with the capacity to train new members in the research base for risk-reduction approaches to prevention.	1. National framework supporting community mobilisation 2. Funding structure for coalition-based prevention activities and technical assistance 3. Step by step process to follow to mobilise community and deliver strategies 4. Coalitions of interested stakeholders and community leaders	• Menu of strategies • Credibility and trust in process • Motivation • Limited flexibility • Organisation and collaboration	1. Funding available at a government or local level for technical assistance and support (context) ➔ credibility and early support for the intervention (reasoning) ➔ positive implementation outcomes 2. Having a menu of strategies (mech resource) ➔ within the context of key community stakeholders and residents on the coalition (context) ➔ limits flexibility but provides structure for the coalition that they can follow and feel secure that it will lead to implementation success ➔ promotes sustainability beyond the trial period (implementation outcomes). 3. The structure and methodology of a programme like CTC (mech resource) ➔ within the supportive societal context of adolescent health prevention (context) ➔ promotes credibility, organisation and motivation (mech reasoning) ➔ for quicker success in implementation and health behaviour outcomes (outcomes).	^***^
Collins et al. 2007 [71] (USA) *Kentucky Initiatives for Prevention (KIP)*	**Description** Kentucky received a $9 million State Incentive Grant (SIG) from the federal Centre for Substance Abuse Prevention. The Kentucky SIG, called the Kentucky Incentives for Prevention (or KIP) Project, had as its goals (a) strengthening the state youth substance use-prevention system, (b) strengthening the prevention systems within local communities that receive funding, and (c) increasing the health and well-being of youth 12 to 17 years of age by reducing posited risk factors, increasing protective factors, and reducing the use of alcohol, tobacco, and other drugs. The SIG grant was awarded by CSAP to the Kentucky governor’s office and was administered by the Kentucky Division of Substance Abuse (DSA). **Coalition composition** 19 coalitions across various counties in Kentucky. **Results** Short-term results (using 8th grade data) showed no significant decreases in six prevalence of substance use outcomes—and, in fact, a significant though small increase in prevalence of use of one substance (inhalants). Sustained results (using 10th grade data), however, showed significant, though small decreases in three of six substance use outcomes—past month prevalence of cigarette use, alcohol use, and binge drinking. We found evidence that the sustained effects on these three prevalence outcomes were mediated by two posited risk factors: friends’ drug use and perceived availability of drugs. Finally, we found that the number of science-based prevention interventions implemented in schools within the coalitions did not moderate the effects of the coalitions on the prevalence of drug use.	1. History of coalition interventions and a strong focus on adolescent health risk behaviour 2. Funding structure to support process	• Menu of strategies • Technical assistance • Organisation • Trust • Low flexibility and ownership	1. Long history of coalition interventions and a strong focus on adolescent health risk behaviour through funding structures (context) ➔ technical assistance, menus of strategies, regularly meetings and prevention science training (mech resource) ➔ limits flexibility and ownership over the intervention as the coalition are restricted to the set of programmes they can deliver (mech reasoning) ➔ promotes organisation and trust in the established programme (mech reasoning) ➔ to successful implementation and eventually beneficial adolescent health outcomes.	^***^
Flewelling et al. 2005 [20] (USA) *New Directions*	**Description** Vermont’s SIG, New Directions ~ ND!, represented a major shift in the state’s approach to substance abuse prevention through its funding of community coalitions rather than individual programmes. The ND project was based on the premise that effective communitywide prevention of adolescent substance use requires coordination among multiple organisations and institutions, encompassing a comprehensive mix of prevention activities. To that end, full-time coalition coordinators were hired and trained in principles of effective community mobilisation in order to increase local sense of community, enhance mobilisation capacity, and increase community readiness for coordinated research-based prevention activities. **Coalition composition** 23 Coalitions across Vermont. Mixture of strategic leaders with political leverage and community residents. **Results** Across the communities served by these coalitions, greater reductions in student substance use prevalence were achieved, relative to the remainder of the state, for all nine substance use measures examined. The greatest relative reductions were observed for past-30-day use of marijuana and cigarettes (both p, .05). These findings suggest that collaborative community-based efforts implemented within a supportive framework such as Vermont’s New Directions project can have a meaningful impact on the prevalence of substance use behaviours among youth.	1. Community readiness processes prior to coalition selection 2. Funding structure on a national and state level 3. Mixture of strategic and community residents 4. Funded technical assistance	• Flexibility • Empowerment • Community and intervention champions • Organisation	1. Considerable time and planning is spent on community readiness (context) ➔ motivation and acceptance will be triggered in the community when the strategies are rolled out (mech resource) ➔ successful implementation and reduction in adolescent health risk behaviours 2. Shift in public health approach (from individual to community-level) (context) ➔ supportive environment and resources with all elements of the system working to ensure the success of the intervention ➔ strong motivation at all levels (mech reasoning) ➔ positive implementation and health risk behaviour outcomes. 3. Funding (context) ➔ ensures training available for coalition (resource) ➔ which maintains motivation leading to successful implementation 4. If community residents select/design strategies that they believe to be the most appropriate for their community (context) ➔ enhanced flexibility and empowerment (mech reasoning) ➔ commitment and motivation from the community to the tailored programmes ➔ positive outcomes 4.1 **RIVAL THEORY:** If community residents select/design strategies that they believe to be the most appropriate for their community (context) ➔ misunderstanding of prevention focus and lack of confidence or ability to design effective strategies ➔ unclear outcomes 5. Community members and key stakeholders are on the coalition (context) ➔ they are able to enhance public visibility of the intervention (from trusted people in the community) and prepare the community for programmes (mech) ➔ leading to positive outcomes out of the collaboration	^***^
Berkley Patton 2004 [72] Paine Andrews 1996 (USA) [73] *Project Freedom (Lawrence)*	**Description** Project Freedom model was funded by the Kansas Health Foundation. Three Kansas sites were awarded grants. Founding participants of the Project Freedom of Lawrence coalition were five community residents, primarily from the local school district, who had come together to write the grant and conduct the pre-planning activities to include in the Project Freedom of Lawrence proposal. $50,000 planning first year, then $100,000 each year for three years for activities. The coalition also generated resources from in-kind donations and cash funding for the support of coalition functioning and institutionalisation. Over the course of four years, the coalition secured $112,000 in funding from various sources (e.g. City of Lawrence alcohol tax funds, Office of Juvenile Justice) and achieved close to 150 units of resources generated, which was primarily in-kind donations. Its aim was to bring about changes in programmes, policies, and practices that would reduce use of alcohol, tobacco and illegal drugs among 12–17 year olds. **Coalition composition** 7 community members on coalition, including young people (but expanded to include hired staff) 273 coalition members in wider cohort. **Results** The Project Freedom of Lawrence coalition successfully replicated the model for community mobilisation (i.e. planning, implementation, evaluation, institutionalisation) used by the original Project Freedom, yet only achieved modest positive outcomes. Project Freedom of Lawrence created 68 community changes over a four-year period. Substance use increased for all drugs in the first two years of implementation (i.e. 1993, 1994). Decreases in the last year of grant funding (1997) were found for any and regular use of tobacco for all participating grades (6th, 8th and 10th graders). In summary, the Project Freedom of Lawrence coalition successfully replicated the model for community mobilisation (i.e. planning, implementation, evaluation, institutionalisation) used by the original Project Freedom, yet only achieved modest positive outcomes compared to the original effort.	1. History of coalition prevention, established framework 2. Community formed in grassroots process and then apply for funds (not already established or part of local authority) 3. Funding structures at local and national level	• Community champions of the intervention • Flexibility, ownership over strategies design and choice • Technical assistance • Motivation due to sustainability	1. If community members (champions) who act as catalysts for the intervention are few (mechanism) ➔ even within a context supportive of community mobilisation approaches like the USA (context) ➔ change and implemented strategies may take longer than planned, as there are no leaders to motivate the community (mechanism reasoning) ➔ leading to poor health risk behaviour outcomes (outcomes). 2. If the coalition starts with community members applying for funds in a grassroots way (context) ➔ the community are able to design and develop their own chosen strategies relevant to the community problem of adolescent health risk behaviours (mechanism resource) ➔ sense of ownership, empowerment and flexibility (reasoning) ➔ ensuring commitment to the intervention and leading to positive community level and health risk behaviour outcomes (outcome). 3. If there is funding available at a government or local level for grassroots organisations or local authorities to apply for (context) ➔ coalition can pay for much needed technical assistance and the delivery of a wide range of strategies (resources) ➔ stronger motivation for the intervention as coalition and community members know there is sustainability in their efforts (mech reasoning) ➔ positive community level and implementation outcomes.	^***^
Fawcett et al. 1997 [74], Lewis et al. 1995 (USA) [75] *Project Freedom (Wichita)*	**Description** Coalition members implemented community initiatives such as store clerk education, Coalition building was the overarching strategy used by Project Freedom, with emphases on coordinating resources and referrals and filling gaps in services. The coalition attempted to serve as a catalyst, not as a service agency. Its aim was to bring about changes in programmes, policies, and practices that would reduce use of alcohol, tobacco and illegal drugs among 12–17 year olds. This required the development of task forces that represented multiple sectors of the community, such as schools, social service organisations, and criminal justice. **Coalition composition** No specific details about the coalition composition. Project Freedom had input from over 100 organisations and individuals in the community. **Results** The largest effects were noted with alcohol; showing reductions in reported regular use from 25.1 to 21.9% in Sedgwick County compared to 25.2 to 23.3% state-wide. More modest effects were noted with marijuana; from 7.5 to 7.1% in Sedgwick County compared to 6.1 to 6.2% state-wide. Similarly small effects were noted with cocaine; from 2.1 to 1.6% in Sedgwick County compared to 2.0 to 1.9% state-wide. Reported regular use of cigarettes increased in Sedgwick County from 24.3 to 25.3%, a slightly higher increase than that observed state-wide (from 22.2% to 22.9%). Smokeless tobacco use increased somewhat in Sedgwick County, from 7.1% to 9.1%, while state-wide reported use decreased from 10.2 to 9.9%. These preliminary findings suggest that implementation of Project Freedom’s action plan, and the community changes that were produced, may have brought about improvements in community- level indicators. Of course, other correlated events that occurred before or during the coalition’s efforts, such as DUI-prevention grants, may have accounted for the observed changes in community-level indicators.	1. City/urban area 2. Pre-existing coalition designed to deal with a range of community issues 3. High levels of adolescent health risk behaviour	• Community champions • Motivation • Commitment to intervention • Split focus between intervention and other community issues • Awareness of community issues related to adolescent health risk behaviours (gang behaviour and substance use) • Knowledge of policy level interventions • Political leverage • Organisation and momentum	1. If within the city context (context) ➔ champions or key leaders who are driving the intervention leave (mech resource) ➔ motivation may slip as there is no catalyst for steady and ongoing commitment to the project (mech reasoning) ➔ poorer implementation outcomes. 2. If the coalition is pre-existing, with a new remit of adolescent health risk behaviours and new members (context) ➔ there may be a split focus and it may be challenging to put energy and motivation into the intervention while there are other community issues (mechs) ➔ leading to slower implementation success and potentially less impact on health risk behaviours (outcomes). 3. Coalitions and community members within a city context (context) ➔ may have greater awareness of substance use and gang related community issues ➔ stronger to enact change in this population (mech) ➔ positive implementation and health risk behaviour outcomes. 4. If the coalition is pre-existing (context) ➔ the members may have greater political and community influence and knowledge about policy-level interventions (resource) ➔ leading to a range of strategies (not just school-based programmes) ➔ enabling greater organisation and momentum to see reductions in adolescent MRB).	^**^
Spoth 2007 [76], 2011 [77] & Chilenski 2016 (USA) [78] *PROSPER*	**Description** A three-component community-university partnership model guided the implementation of evidence-based interventions (EBIs), as described in detail previously. The three components of the PROSPER model consist of local community teams, state-level university researchers, and a Prevention Coordinator team in the land grant university Cooperative Extension System. Prevention Coordinators served as liaisons between the community-based teams and university researchers, providing continuous, proactive technical assistance to the community teams. **Coalition composition** 8–12 on each community team, across 28 school districts in Iowa and Pennsylvania. Community teams were comprised of a Cooperative Extension staff team leader, a public school representative co-leader, and representatives of local human service agencies, along with other local community stakeholders (e.g. youth and parents). **Results** Results showed significantly lower substance use in the intervention group for 12 of 15 point-in-time outcomes, with relative reductions of up to 51.8%. Growth trajectory analyses showed significantly slower growth in the intervention group for 14 of 15 outcomes.	1. Established funding structure on a national and state level 2. History of established method for prevention in coalition and community work 3. Mixture of strategic, community and youth partners	• Motivation • Awareness of sustainability • Menu of evidence based strategies (confidence) • Trust and credibility of the intervention process • Limited flexibility • Focus on the strategies	1. If there is funding available at a government or local level (context) ➔ coalition can pay for much needed technical assistance and the delivery of a wide range of strategies (resources) ➔ stronger motivation for the intervention as coalition and community members know there is sustainability in their efforts (mech reasoning) ➔ positive community level and implementation outcomes. 2. A long history and established method for adolescent health coalitions (context) ➔ a menu of strategies and school-based programmes (mech resource) ➔ promotes trust among coalition and community members (reasoning) ➔ leading to successful implementation 3. A long history and established method for adolescent health coalitions (context) ➔ a menu of strategies and school-based programmes (mech resource) ➔ limits flexibility and empowerment of the coalition members (reasoning), ➔ the focus is largely on the evidence based programmes to deliver successful outcomes, not the community members and issues ➔ whatever they choose has been evaluated and should lead to positive outcomes if implemented correctly	^***^
Brown-2017 [79] (Mexico) Red de Coaliciones Comunitarias de Mexico (The Network of Community Coalitions in Mexico)	**Description** The model employed to train the coalition members to develop their action plans was the Strategic Prevention Framework (SPF), which is a cyclical 5-step process for implementing sustainable evidence-based practices to prevent youth substance use. The Community Anti-Drug Coalitions of America (CADCA) provided training that emphasised comprehensive action using seven individual and environmental change strategies—(1) provide information, (2) enhance skills, (3) provide support, (4) enhance access/reduce barriers, (5) change consequences, (6) change physical design and (7) change policies. Thus, rather than drawing from a list of evidence- based programmes, the coalitions were taught how to identify strategies that could address the fundamental causes and local conditions behind the community problems prioritised in their community diagnosis. Coalitions sought community and population level changes through the comprehensive implementation of best practices for the seven community change strategies. Coalitions were provided with $10,000 per year for activities. **Coalition composition** Approximately 316 individuals over 17 coalitions, made up of strategic partners, community residents and youth. **Results** Coalition functioning outcomes. The results failed to support the first hypothesis that sectoral diversity would have a positive impact on coalition outcomes of community support for the coalition, community improvement from coalition activities, and coalition sustainability planning. This null result may be in part because at the time of the study these coalitions were only 1–3 years old. Although sectoral diversity was not associated with coalition outcomes, higher levels of intersectoral communication were, in two of the three outcome measures. Coalitions with members who sought advice from more members in other sectors perceived higher levels of support by community leaders and organisations. If Mexican coalitions want to address problems with a lack of effective and trustworthy law enforcement, the strategies used will need to be vastly different from those taken by drug prevention coalitions in the United States, which often focus on implementing school- and community-based programmes [62].The Mexican coalitions also face lower levels of support for community prevention activities and fewer community champions, making it challenging to build the momentum necessary to take action. Mexican coalitions may need to focus additional energy on building community support for their implementation efforts to be successful. However, this push to move forward is in some tension with the current study, which implies that coalition leaders may need more time to build a collective identity and to create and solidify communication practices.	1. Distrust between community and law enforcement 2. Lack of prevention and intervention delivery structure 3. Community level problems connected with adolescent risk behaviours (e.g. drug use in family parks)	• Lack of community champions • Split focus • Lack of understanding of prevention science • Ownership (over designing own strategies)	1. If there is a lack of trust between community and law enforcement and lack of a prevention structure (context) ➔ community champions not be fired (mech resource) ➔ making it challenging to build momentum and take action towards adolescent health risk behaviours 2. Community-level problems related to adolescent health risk behaviours (e.g. drug use in family parks) (context) ➔ community residents motivated to design their own strategies to change the physical environment (resource) ➔ positive coalition functioning as coalition members have a sense of ownership over the intervention ➔ this does not necessarily lead to positive health risk behaviour outcomes and may result in a split focus on community issues (mech)	^***^
Cheadle et al. 2001 [80] (USA) *Minority Youth Health Project*	**Description** The intervention consisted of a paid community organiser in each neighbourhood who recruited a group of residents to serve as a community action board. Key variables included perceptions of neighbourhood mobilisation by youth, parents, and key neighbourhood leaders. **Coalition composition** A mix of both strategic partners and community residents across 4 communities. Unclear size of coalitions. **Results** The MY Health Project did not produce a measurable effect on community mobilisation in the four neighbourhoods where intervention activities were carried out. It is uncertain whether this was because of a lack of strength of the interventions or problems detecting intervention effects using the surveys available. The uncertainty underlying these results provides further support for the argument that large-scale randomised trials may not be the best way of evaluating community-based health interventions. Community mobilisation activities may not have been strong enough relative to the size of the neighbourhoods to produce visible, population-level changes. The mobilisation campaign took longer than expected to implement, partly because of a change in strategy from using existing neighbourhood leaders to a more grass roots approach. In addition, because they were new to serving on organised community boards, the CAB members may have taken longer to become organised and choose projects to implement.	1. Seven city co-operative agreement (urban areas) 2. Grassroots approach (new coalition created) 3. Lack of oversight structure and no established framework 4. Unsure of community readiness levels (no assessment done) 5. Provided grants for activities and staff to assist	• Community empowerment • Community pride and identification with neighbourhood • Flexibility and community ownership over intervention activities	1. Grassroots approach whereby coalitions are formed at the start of the trial ➔ flexibility to implement a wide variety of activities ➔ differing levels of success between neighbourhoods and challenge to measure the effects in an RCT ➔ unclear about improvements in health risk behaviours 2. Community readiness activities not strong enough relative to neighbourhood size ➔ low community empowerment and neighbourhood identification ➔ weak community coalitions, no community champions and low trust in intervention activities ➔ null health risk behaviour outcomes 3. Urban areas chosen as part of participation in collaboration and proportion of youth of colour (not community readiness measures) ➔ community boundaries not clear, low identification with neighbourhoods ➔ challenging to measure differential effects between neighbourhoods 4. Provided grants for activities and staff to assist ➔ motivated to implement activities in the community ➔ potential for positive implementation and health risk behaviour outcomes 4.1 **RIVAL THEORY:** Provided grants for activities and staff to assist (without training on sustaining the coalition) ➔ decisions and oversight still lie with community residents, leading to a lack of structure and focus on prevention activities ➔ difficult to measure the implemented strategies and most disbanded after the trial period.	^***^
Keene Woods et al. 2014 [81] and Keene Woods 2009 [82] thesis (2 documents to extract from) *The Youth Community Coalition (YC2)*	**Description** The Youth Community Coalition (YC2) of Columbia, Missouri, was formed in 2003 by the Columbia Housing Authority to address local community needs. In 2004, the coalition began to focus on adolescent substance use prevention after receiving funding from the Drug-Free Communities Program grant. In 2009, the coalition was selected to participate in the NIDA Coalition Research Project (NCRP) funded by the National Institute on Drug Abuse of the National Institutes of Health, as part of a larger research study. The coalition was not provided funding as part of the study, receiving only the intervention and travel reimbursement for participation. The Community Change Intervention consisted of two components: (a) in-person training in core competencies using a field-tested curriculum and (b) tele- phone-based TA for implementing priority key processes identified by the coalition. **Coalition composition** Over 70 organisations and individuals represented across the coalitions. Mixture of strategic partners and community residents. The coalition was already active but was then selected for training and implementing key processes for youth substance use. **Results** Over the 2-year intervention period, there were 36 community changes facilitated by the coalition to reduce risk for adolescent substance use. Results showed that the coalition facilitated an average of at least 3 times as many community changes (i.e. program, policy and practice changes) per month following the intervention. Action planning was found to have accelerated the rate of community changes implemented by the coalition. After the intervention there was increased implementation of three key prioritised coalition processes: Documenting Progress/ Using Feedback, Making Outcomes Matter, and Sustaining the Work. A 1-year probe following the study showed that the majority of the community changes were sustained. Factors associated with the sustainability of changes included the continued development of collaborative partnerships and securing multiyear funding.	1. Pre-existing coalition selected for additional purpose/training 2. Framework and funding for coalition-based prevention work in communities 3. Funding provided to coalition for training and to deliver change 4. Oversight body and established process	• Motivation • Trust and credibility of coalition model • Organisation • Focused on a common goal • Confidence based on experience working on coalitions • Low flexibility	1. Pre-existing community coalition selected for additional adolescent health risk behaviour training ➔ strong focus on the goal of reducing adolescent health risk behaviours using prevention science ➔ organisation and trust ➔ positive community changes and implementation outcomes 2. Pre-existing community coalition selected from a societal framework of 5000 coalitions ➔ trust in the coalition method and credibility in its members ➔ positive community changes and implementation outcomes 3. Funding provided to coalition members for training and to deliver change ➔ motivation around a common goal ➔ positive community changes and implementation outcomes 4. A framework and resources provided by oversight body ➔ trust, capability and motivation to complete the framework (low flexibility) ➔ positive community changes and implementation outcomes	^***^
Shaw et al. 1997 [83] (USA) *The Gloucester Prevention Network*	**Description** The Gloucester Prevention Network is a comprehensive community ATOD prevention partnership with multiple coordinated community prevention activities, including several peer education programmes in the schools. **Coalition composition** 10 thematic coalitions working towards one goal. Mixture of strategic partners and community residents. Coalitions had already been working for some time. **Results** The results suggest that the comprehensive coalition approach was effective for all substance use outcomes with the exception of marijuana use. A number of coalitions developed peer educator programmes in schools, which were seen as key elements in modifying young people’s behaviour towards alcohol and other drugs. There was a decrease in alcohol behaviour for boys but not girls, which is not easily explained. Boys were more involved in youth sports leagues so the prevention programmes there may have led to a greater impact on young men in the community. Gender differences in health risk behaviours were not accounted for in intervention delivery. Normative changes in community systems reinforced changes in students’ behaviour and attitudes. Many community events were alcohol free and tougher driving while intoxicated laws were enforced. Religious coalitions and local businesses were involved in initiatives. These community strategies created a climate where changes in adolescent behaviours were more likely to occur.	1. High rates of adolescent health risk behaviours 2. Societal context that supports public health as a delivery system 3. Focused effort to include youth in intervention from the start 4. Wider structural and financial framework for preventing adolescent health risk behaviours 5. Coalition mixture of strategic partners and community residents 6. Youth consultation approach	• Systems approach (weaving prevention into community issues) • Ownership and collaboration • Empowerment among youth • Tailoring of strategies • Prevention science training • Trust in sustainability (due to funds)	1. High rates of adolescent health risk behaviours AND the societal context that supports public health as a delivery system (context) ➔ a multi-level or systems approach can be triggered (mech resource) including multiple activities that provide the environment for young people to change their behaviour ➔ ownership and collaboration within the community as prevention is woven into all community issues (e.g. events being alcohol free) ➔ successful implementation resulting in positive community and health risk behaviour outcomes. 2. High rates of adolescent health risk behaviour AND youth are heavily involved in the intervention activities through consultation (context) ➔ empowerment among young people and specifically tailored strategies (mech) ➔ positive implementation outcomes and reductions in adolescent health risk behaviours (outcomes). 3. A wider structure (financial and societal) geared towards reducing adolescent health risk behaviours (context) ➔ prevention science training can be provided and decisions are made on empirical adolescent public health data (mech) ➔ empowerment within the community as the funds are there to tackle the problem (reasoning) ➔ positive outcomes. 4. Within the context of deprived communities with high levels of health risk behaviours (context) that there are already gender differences within health risk behaviours (e.g. boys having higher rates of substance use than girls) ➔ sports clubs delivered without gender considerations ➔ greater reductions in boys’ health risk behaviour engagement.	^**^

This table displays the intervention name, a summary of the intervention content, coalition information and results reported in any evaluation documents, the contexts and mechanisms identified by the researchers, selected examples of CMO configurations applicable to each intervention and the quality assessment score

## Main findings

### Programme theory and middle range theories

As was expected due to the complexity and heterogeneity of approaches within community mobilisation interventions, it was a challenge to determine a single programme theory. Based on the previous literature explored in the background of this article and discussions with stakeholders, we determined a broad, overarching programme theory that for community mobilisation interventions to result in reductions in adolescent MRB: interventions must explicitly seek to affect community-level influences through coalition-led initiatives, by creating social environments that mean that adolescents are less likely to engage in risky behaviours.

Under the umbrella of our broad programme theory, we produced a synthesis of three middle range theories describing the various CMO configurations that explain elements of how these interventions work or do not work. Through critically reflecting on these middle range theories, which we have presented thematically below, we answer our research questions of which contextual and mechanistic factors are present within our sample to produce outcomes.

Our three middle range theories developed as contributing to our understanding (our overarching programme theory) of how the community mobilisation interventions ‘work’ are:

1. Community mobilisation interventions achieve positive adolescent MRB outcomes when supported by guiding principles and the focus is on collaborating across factions of society.
2. Community mobilisation interventions achieve positive adolescent MRB outcomes when the community readiness is established through understanding the community social norms and prevalence through population level data, which motivates the community to create an environment for prevention.
3. Community mobilisation interventions achieve positive adolescent MRB outcomes when the community coalition has diverse membership with expertise and community members, triggering empowerment, support and knowledge.

Not all studies contribute to our thinking around the middle range theories and therefore not every CMO configuration contains every study, if there was no evidence within the study. Further, there are times where interventions report positive outcomes, but we have interpreted that a mechanism in question has not been ‘triggered’. Therefore, in the analysis tables where studies are listed under ‘Mechanisms not triggered’, this is not to say that they are not potentially successful and useful interventions, but simply that we did not interpret them to be adhering to that particular CMO configuration. Throughout the text (C), (M) and (O) labels appear to highlight aspects we interpreted as contexts, mechanisms and outcomes, to add clarity and enhance transparency of our analysis.

#### Middle range theory 1—Community mobilisation interventions achieve positive adolescent MRB outcomes (O) when supported by guiding principles (C) and the focus is on collaborating across factions of society (M)

While community mobilisation interventions aim to address the community-level causes of adolescent multiple risk behaviour, only some of the included studies appeared to be committed to that goal. The community mobilisation interventions that had the greatest success in terms of ongoing implementation and prevention of adolescent MRB tended to focus on embedding the intervention ‘principles’ throughout multiple factions of the community (e.g. schools, family, wider community/neighbourhood) (C). In these cases [20, 23, 51, 52, 63, 74, 84], the intervention is a framework, model or way of thinking, framed by ‘guiding principles’ as opposed to one-off programmes that need to be delivered in a certain way. This approach allows support and a way to structure collaborative efforts, without being too prescriptive, instilling empowerment for coalition members (M) to choose how to deliver the intervention within a proven framework [22].

Moreover, by taking the focus away from individual behaviour change or delivering school-based educational programmes and instead focusing on encouraging coalition and community cohesion through establishing strong collaboration and strengthening connections between family, schools and the wider community (C) [50], these seven interventions were able to deliver multi-level strategies and all work towards the same goal of social environmental change. This approach, over time, resulted in reductions in adolescent MRB in their community (O). The reason they were successful, we believe, is that they were more closely aligned with an environmental and systems way of thinking, instead of trying to teach young people not to engage in MRB, which is increasingly known to be largely ineffective and potentially increasing of inequalities [85]. If the intervention’s ‘guiding principles’ are woven throughout schools, policy areas including health and education, communities and families (C), then the societal shift needed to prevent adolescent MRB will come from the ‘bottom up’ and is more likely to be directly relevant to the community trying to be changed. It also brings different areas of the community in even if they do not sit directly on the coalition (e.g. school leaders, church leaders), as changing the social environment requires collaborative participation of a wide range of community members (M) [22]. Details of ‘outreach’ activities used to instil the guiding principles and encourage collaboration appeared infrequently within the data beyond note of ‘informalised collaborative activities’ [20], but some included meeting with city and parks personnel and making phone calls to elected officials [74] and supporting grassroots organisations, and partnerships with police [23].

Mechanistically, having a set of ‘guiding principles’ (C) encourages empowerment (M) among coalition and community members (where an instructive intervention pack would not), through the ability to design and deliver relevant programmes. The coalition can constantly revisit the principles when making decisions [22]. Some studies contradicted this theory in that they still managed to result in positive health risk behaviour outcomes in the absence of this context and mechanism combination. For example, interventions in the USA such as Communities that Care (CTC) [55], PROSPER [76] and Community Prevention and Wellness Initiative (CPWI) [53] appeared to be focused on a core component of the compulsory delivery of evidence-based (usually school-based) programmes with less apparent focus on the establishing collaborative networks (C). Although these studies do begin with a framework for intervention delivery, the essential element of collaboration and creating environmental shifts appeared to be less important than selecting from a ‘menu’ of evidence-based programmes (EBPs) [57, 76]. In contrast, the CTC programme in Australia had stronger focus on the collaborative approach and systems way of thinking (M) and less on which specific types of programmes were delivered [63]. For this reason, we determined that some studies strongly aligned with this middle range theory [20, 23, 51, 52, 63, 74, 84], but not others [53, 55, 59, 72, 76, 83], despite all seeing reductions in health risk behaviour outcomes.

We found that other, less successful, interventions were either focused more on simply choosing and delivering programmes as opposed to establishing long-term connections (M) [67–69] or were embedded within in societal contexts that made it difficult to strive for a systems approach (C) [54, 79]. These interventions appeared to be less successful in creating that social environmental shift needed for achieving positive intermediate or health risk behaviour outcomes (O). It was a challenge to fully elucidate the causal pathway with this part of the theory as most studies did not go into detail about the extent to which they adhered to the guiding principles and how exactly they applied the principles to their own decision-making (Table 3).

**Table 3:** CMO configuration 1

		**Supporting evidence**
**Context**	Model of guiding principles integrated into all fractions of adolescent life (e.g. schools, policy, community, family)	**Mechanism triggered** (1) Icelandic Prevention Model [84] (2) Icelandic Prevention Model delivered in Chile [51] (3) Icelandic Prevention Model delivered in Lithuania [52] (4) Communities that Care Australia [63] (5) Fighting Back (2002) [23] (6) New Directions (2005) [20] (7) Project Freedom (Wichita) [74] **Mechanism not triggered** (1) Connect to Protect (C2P) Thailand [54] (2) Communities that Care UK [67] (3) Communities that Care Scottish Pilot [68] (4) Communities that Care Pennsylvania (2015) [55] (5) Communities that Care (CYDS) (2009) [59] (6) Community Prevention and Wellness Initiative (CPWI) (2020) [53] (7) TOGETHER CTC Oregon (1992) [69] (8) Kentucky Initiatives for Prevention (2007) [71] (9) Project Freedom Lawrence (2004) [72] (10) Red de Coaliciones Comunitarias de Mexico [79] (11) PROSPER (2007) [76] (12) The Gloucester Prevention Network (1997) [83] (13) Communities that Care (The Netherlands) [64]
**Mechanism**	Empowerment, support and collaboration—being able to choose what works best for different areas of communities while also being able to stick to a set of principles. Working together across society on a set of principles leading to strong community collaboration.	
**Outcome**	Positive risk and protective factor outcomes and health risk behaviour outcomes through creating an environment where all domains of the community are working towards health risk behaviour prevention.	

The table shows an example CMO configuration with a list of interventions that provide supporting or contrasting evidence of that configuration

We further interpreted, unsurprisingly, that in order to facilitate system-wide prevention of adolescent MRB, there needs to be a strong and supportive funding infrastructure (C). We found this to be a crucial context important for triggering mechanisms such as collaboration, flexibility and motivation (M) from the coalition members and wider community (participants) to continue with the intervention. This CMO configuration was supported by evidence from 12 studies, which reported positive implementation or coalition functioning [55, 59, 69, 72, 76] and/or health risk behaviour outcomes [20, 22, 23, 51–53, 55, 59, 71, 72, 76, 83, 84]. It is not enough to simply have the ‘model of guiding principles’ context, but this must be coupled with long-term, flexible funding that accounts for both technical staff assistance and differing strategies the coalition may implement [50]. Further, the environmental strategies likely to have the most success (e.g. paying for young people to engage in health promoting activities [22], scholarship programmes for at risk youth [74], policy changes around alcohol or tobacco smoking [23]) are also likely to be the most expensive comparative to school-based education programmes. This contextual condition was further highlighted through examples of interventions that had been successful in one context but then had failed to work in the same way in other contexts/countries. Namely, Communities that Care and the Icelandic Prevention Model were both adapted for other contexts (e.g. CTC in the UK [67] and IPM in Chile [51, 52]) and all highlighted funding longevity as a significant barrier to success. Further, the Connect to Protect programme in Thailand [54] did report a commitment to shifting the social environment, but the context they were working in (e.g. significant methamphetamine problems within the community, funding issues, legal approach to drug use, limited community involvement) had contextual factors that made it extremely difficult to deliver the intervention [54]. Our second core result is therefore that context is crucially important to achieving seeing reductions in adolescent MRB from community mobilisation interventions and a condition on this middle range theory (Table 4).

**Table 4:** CMO configuration 2

		**Supporting evidence**
**Context**	Long-term funding structure that supports coalition prevention efforts	**Mechanism triggered** (1) Icelandic Prevention Model (IPM) [84] (2) Community Prevention and Wellness Initiative (CPWI) (2020) [53] (3) Fighting Back (2002) [23] (4) Communities that Care Pennsylvania (2015) [55] (5) Communities that Care (CYDS) (2009) [59] (6) TOGETHER CTC Oregon (1992) [69] (7) Kentucky Initiatives for Prevention (2007) [71] (8) New Directions (2005) [20] (9) Project Freedom Lawrence (2004) [72] (10) PROSPER (2007) [76] (11) The Youth Community Coalition (YC2) (2011) [82] (12) The Gloucester Prevention Network (1997) [83] **Mechanism not triggered (within certain context)** (1) Connect to Protect (C2P) Thailand [54] (2) Communities that Care UK [67] (3) Communities that Care Scottish Pilot [68] (4) Minority Youth Health Project [86] (5) Icelandic Prevention Model delivered in Chile [51]
**Mechanism**	Technical assistance resources activated and sustainability	
**Outcome**	Positive implementation and coalition functioning outcomes (leading to promising adolescent health risk behaviour outcomes)	

The table shows an example CMO configuration with a list of interventions that provide supporting or contrasting evidence of that configuration

#### Middle range theory 2—Community mobilisation interventions achieve positive adolescent MRB outcomes (O) when the community readiness is established through understanding the community social norms and prevalence through population level data (C), which motivates the coalition and community to create an environment for prevention (M)

“Community readiness” repeatedly arose as an important concept [53, 54, 57, 72] and is widely discussed in the community prevention literature [58, 87]. Community readiness refers to a community’s ‘levels of awareness of the problems which they need to address, their perceptions, expectations, and interests related to the problem, their judgments and necessary decision-making processes as well as capabilities, the necessary human and non-human resources to implement effective prevention interventions, and their readiness to initiate prevention activities and to institutionalise and continue them if and when necessary.’ ([87], p.1084). Put simply, if the social environment of the community is going to be changed to support adolescent health (O), the coalition need to know which communities are in most need, which risk and protective factors require the greatest attention and whether the community will accept programmes and in what form (C) [22].

An important part of community readiness in terms of MRB interventions was that the local community had social norms and values compatible with prevention interventions as well as a concern for adolescent health (C) [50]. For example, for the intervention to be successful, the local community and coalition members would need to (1) be concerned about adolescent MRB within their community (e.g. through experiencing high local substance use or violence prevalence rates) and (2) have community and societal social values amenable to a prevention approach to these problems. It is only through these contextual ‘community readiness’ factors that the motivation mechanism would be fired among both the coalition members driving intervention delivery and community members receiving the intervention (M) (Table 5).

**Table 5:** CMO configuration 3

		**Evidence**
**Context**	(1) Concern locally about adolescent MRB *and* (2) social values accepting of a prevention approach	**Mechanism triggered** (1) Icelandic Prevention Model [22] (2) Community Prevention and Wellness Initiative (CPWI) [53] (3) Communities that Care Australia [63] (4) TOGETHER! Communities for Drug Free Youth [69] (5) Kentucky Initiatives for Prevention (2007) [71] (6) New Directions (2005) [20] (7) Project Freedom Lawrence (2004) [72] (8) PROSPER (2007) [76] (9) The Youth Community Coalition (YC2) (2011) [82] (10) The Gloucester Prevention Network (1997) [83] **Mechanism not triggered** (1) Connect to Protect (C2P) Thailand [54] (2) Red de Coaliciones Comunitarias de Mexico (The Network of Community Coalitions in Mexico) [79] (3) Minority Youth Health Project [86]
**Mechanism**	Motivation to tackle MRB among coalition members and community receiving intervention	
**Outcome**	Positive implementation outcomes leading to a reduction in MRB	

The table shows an example CMO configuration with a list of interventions that provide supporting or contrasting evidence of that configuration

Through this CMO, we see that it is not only contextually important that adolescent health risk behaviours are prevalent, but that their framing and discursive representation is also crucial. For example, if adolescent MRB is not viewed as a problem for the community to try and tackle, or if it is viewed as a legal issue (e.g. drug use and violence) (C), then there will be little interest in the community or local stakeholders in pursuing a prevention approach (M). This particular example was highlighted in a included studies in Thailand [54] and Mexico [79], which referenced the established legal frameworks for tackling adolescent substance use as a major challenge to getting the community and stakeholders unified within the intervention, with substantial distrust of government agencies (C). Therefore, the wider context surrounding adolescent health risk behaviours is important for triggering the level of commitment (M) from community members to take part in the intervention. In turn, if the community are committed to the issue and the coalition can galvanise the community, it is expected that positive implementation and health risk behaviour outcomes would follow (O).

Using population data was critical for both establishing community readiness through assessing the size and nature of the problem of adolescent MRB and allowing the coalition and community to be actively involved in data collection/analysis (C). Population data was also crucial for tracking the intervention over a long time period, something mentioned by some of our stakeholders and highlighted in a number of studies [51–53, 59, 63, 64, 69, 84]. To be clear, this is beyond simply evaluating the intervention using survey data or evaluation methods (which all studies did), but is about using population and/or epidemiological data as a the ‘foundation’ of the community mobilisation effort (C) [69]. This approach is also needed for guiding the coalition in establishing what kinds of programmes, at what levels, they may choose to implement [22].

Studies that did not use initial data analysis of adolescent MRB and other related risk and protective factors as its foundation, had challenges doing so or did not mention it as a major part of ‘readiness’ (C) [23, 54, 67, 68, 76, 82], we interpreted, were less able to design strategies uniquely matched to the needs of that community (M). These studies saw the potential value of community mobilisation interventions and delivering events and programmes for the community (and some mentioned the goal of environmental change [23, 54]), but did not take the time to understand how these programmes might bring about long-term change. Without meaningful population-level data at initiation and a commitment to tracking that data throughout, mechanisms including flexibility (e.g. the ability to react to evidence coming from the community) (M) can lose sight of the intervention aims [22]. This issue was raised by one study, highlighting that while they were aiming to empower community members by giving them flexibility (M), in practice it meant that ‘activities were only indirectly related to youth health behaviours’ (O) [80], whereas greater engagement with population data would have resulted in strategies more directly connected to the intervention objectives (Table 6).

**Table 6:** CMO configuration 4

		Evidence
Context	Using population data to establish community readiness, make decisions and track progress	**Mechanism triggered** (1) Icelandic Prevention Model [22] (2) Icelandic Prevention Model delivered in Chile [51] (3) Icelandic Prevention Model delivered in Lithuania [52] (4) Community Prevention and Wellness Initiative (CPWI) [53] (5) Communities that Care Australia [63] (6) TOGETHER! Communities for Drug Free Youth [69] (7) New Directions (2005) [20] (8) Project Freedom (Wichita) [74] (9) Communities that Care (CYDS) (2009) [59] (10) Communities that Care Pennsylvania [55] (11) Communities that Care (The Netherlands) [64] **Mechanism not triggered** (1) Connect to Protect (C2P) Thailand [54] (2) Red de Coaliciones Comunitarias de Mexico (The Network of Community Coalitions in Mexico) [79] (3) Minority Youth Health Project [86] (4) Communities that Care (Scottish Pilot) [68] (5) Communities that Care (UK) [67] (6) Kentucky Initiatives for Prevention (2007) [71] (7) PRSOPER [76] (8) Minority Youth Health Project [80] (9) The Youth Community Coalition [82] (10) The Gloucester Prevention Network (1997) [83]
Mechanism	Local knowledge of the community, flexibility to adapt to the data, motivation to continue by seeing the impacts and being involved in data analysis.	
Outcome	Changes in the social environment leading to reductions in adolescent MRB.	

#### Middle range theory 3—Community mobilisation interventions achieve positive adolescent MRB outcomes (O) when the community coalition has diverse membership with expertise and community members (C), triggering empowerment and support (M)

We identified that the *coalition composition* was an important resource (C) that determined whether certain reactive mechanisms were triggered in particular contexts. Across the documents, we found there to be limited detail about the makeup of the coalitions involved in the interventions. Where we were able to extract data, however, we inferred from the data that the coalitions most likely to create an environment where adolescents are less likely to engage in risky behaviours were those that achieved a balance of expertise (e.g. civil servants, policymakers, social scientists) and community relevance (e.g. community members, parents, young people) through coalition membership. We determined that focusing on getting the right configuration of the coalition (C) enacted the mechanisms of empowerment, knowledge and ability (e.g. ‘political leverage’ [20]) (M) needed to alter the environment (O).

Although there were limited details regarding the coalition configurations in most studies, from what we could glean, the data suggested that most interventions attempted to form a coalition that had a mixture of expert stakeholders and community members [20, 23, 53, 54, 59, 63, 64, 67, 74, 76, 79, 84, 82]. The study that had purely civil servants on the coalition [69] and the study that was made up entirely of community members [72] both faced challenges in implementation and achieving prevention outcomes, which they partially attributed to the coalition functioning. Critically, several studies tried to embrace the mixture of expertise and community members (C) yet had significant issues during implementation (M) or did not report community reductions of adolescent MRB (O) [54, 64, 69, 79, 80] (See Table 7). For example, for the Communities that Care in the Netherlands [64], there was a challenge in engaging the right mix of people, with the coalitions being made of up of representatives from local institutions working with children, who often left when they moved job role. They noted a distinct lack of participation from ‘students, business leaders and volunteers [community members]’ [64] (C), which they found to be a barrier to achieving the right knowledge and community attachment (M) needed to make environmental changes for adolescent health (O). Further, Brown et al. noted that lower community support and lack of collective identity (C) within the coalitions in the Red de Coaliciones Comunitarias de Mexico study [79], led to ‘lack of momentum necessary to take action’ (M).

**Table 7:** CMO configuration 5

		**Evidence**
**Context**	Coalition with a mix of expertise (policy, social science and research stakeholders) and community members	**Mechanism triggered** (1) Icelandic Prevention Model [50] (2) Community Prevention and Wellness Initiative (CPWI) [53] (3) Communities that Care Australia [63] (4) TOGETHER! Communities for Drug Free Youth [69] (5) New Directions (2005) [20] (6) Project Freedom Lawrence (2004) [72] (7) PROSPER (2007) [76] (8) The Youth Community Coalition (YC2) (2011) [82] **Mechanism not triggered** (1) Communities that Care in the Netherlands [64] (2) Red de Coaliciones Comunitarias de Mexico (The Network of Community Coalitions in Mexico) [79] (3) Connect to Protect (C2P) Thailand [54] (4) Minority Youth Health Project [86] **No information on coalition configuration** (1) Communities that Care Scottish Pilot [68] (2) Icelandic Prevention Model Chile [51] (3) Icelandic Prevention Model Lithuania [52] (4) Kentucky Initiatives for Prevention (2007) [71] (5) The Gloucester Prevention Network (1997) [83]
**Mechanism(s)**	Empowerment from within the community and knowledge and ability (leverage) to enact change and collaborate across society	
**Outcome**	Creating shifts in the social environment towards prevention of adolescent MRB	

The table shows an example CMO configuration with a list of interventions that provide supporting or contrasting evidence of that configuration

We determined that for this middle range theory to be upheld and result in positive outcomes, additional components are needed so that the coalition part of the intervention can function successfully. *Intervention champions* were interpreted as an important contextual feature (C) within the IPM [50], Communities that Care (CYDS) [59], New Directions [20], Project Freedom (Lawrence) [72] and Project Freedom (Wichita) [74] and was mentioned by a stakeholder describing the implementation of Communities that Care Australia [63]. These champions speak about the intervention as much as possible, as a formal part of their responsibilities. It becomes a major part of their role to advocate for the intervention, so it remains on the agenda for public health prevention, schools and other agencies. These champions are a particularly crucial for triggering collaboration and establishing connections across different domains of the community (M). Crucially, two studies [79, 80] highlighted that *not* having community champions (C) meant they had a reduced ability to create the collaborative networks (M) needed to improve the environment for adolescent health (O) (Table 8).

**Table 8:** CMO configuration 6

		**Evidence**
**Context**	Intervention champions (resource)	**Mechanism triggered** (1) Icelandic Prevention Model [50] (2) Communities that Care (CYDS) [59] (3) New Directions [20] (4) Project Freedom (Lawrence) [72] (5) Project Freedom (Wichita) [74] **Mechanism not triggered** (1) Red de Coaliciones Comunitarias de Mexico (The Network of Community [79] Coalitions in Mexico (2) Minority Youth Health Project [86]
**Mechanism(s)**	Cohesion, trust and collaboration across the community	
**Outcome**	Creating shifts in the social environment towards prevention of adolescent MRB	

The table shows an example CMO configuration with a list of interventions that provide supporting or contrasting evidence of that configuration

Although only highlighted by five studies, we found that intervention champions helped establish community-wide understanding and trust (M). They also kept strategic focus on the intervention and acted as a catalyst from cross-community collaboration [59]. Intervention champions would not necessarily be needed for single component educational programmes. However, for community mobilisation interventions with the goal of joining up different areas of community and policy, they are an essential glue that allows social environmental shifts. They can communicate what is and is not working across the communities and work to continually embed the principles for reducing adolescent MRB (i.e. creating health promoting environments) across schools, policy and neighbourhoods. We present this evidence within this middle range theory as we found that intervention champions essentially bolster the coalition. Intervention champions alone cannot achieve the reductions in adolescent MRB without a strong and diverse group of experts and community members to support them (C). Finally, something missing from the data was mention of involving young people in the coalition (C). This might be happening, but not reported, as one of our stakeholders claimed this was a core element of CTC Australia but there was not a description in the document [63]. However, we had expected to see greater presence of young people within the community mobilisation interventions but given the lack of data on this element this is not something we have been able to explore using CMOs in the present synthesis.

## Discussion

This realist review synthesis identified important contextual and mechanistic factors that are critical to the successful implementation and effectiveness of community mobilisation interventions for adolescent multiple risk behaviour. The central finding of our review is that for community mobilisation interventions to ‘work’, they must be committed to evoking a set of ‘guiding principles’ that coalitions can use to deliver the intervention their own way. One framework that has been particularly successful in this regard is Icelandic Prevention Model (IPM) [84], with this model becoming increasingly popular and several countries either planning to implement it or exploring it as an option [88–90]. However, we should note the critiques surrounding IPM due to the lack of clarity on successful intervention components and few studies being able to establish causal links to health outcomes [51, 91]. This was reflected in our sample as only three documents measured outcomes [51, 52, 84] despite the intervention having been run for many years across the world [51]. Therefore, although we inferred success in these interventions and attributed that partially to the adherence to guiding principles and social environmental change, we present this finding with caution due to the lack of outcomes papers and the challenge in establishing whether it was the intervention or some other environmental factor (e.g. Covid-19 pandemic [51], policy or societal changes [84, 92]) that resulted in the outcomes.

We further found that the context of the delivery of these interventions is crucially important. The importance of context is illuminated by commentary documents relating to the delivery of the *Communities that Care* (CTC) model in countries outside the USA. For instance, Basic [87] describes the community readiness-related barriers to intervention success in Croatia. Low awareness among stakeholders of the problems of adolescent health risk behaviours, as well as a lack of realistic assessment of available and accessible resources for initiating and sustaining changes, hindered the progress of CTC. Perez-Gomez et al. [93] saw promise in CTC for middle- and low-income countries in Latin America, but their pilot in Colombia conveyed that time and funding must be spent to establish community readiness among residents but also local authority personnel. There was often initial interest in the intervention from local authorities, but a reluctance to make financial commitments to the programmes and at times a lack of motivation to drive the intervention [93]. Further, for IPM, a commentary by Koning et al. [91] highlighted how the context of Iceland is particularly suited to the intervention and warned against other countries trying to replicate without strong consideration of context.

These examples reiterate that even with a proven community mobilisation intervention like CTC or IPM, the wider context that supports intervention delivery (e.g. funding, local authority buy-in, community understanding of the problem) affects the trust, motivation and ability of potential coalition members, which are essential mechanisms for sustaining the intervention to achieve positive outcomes. Although this finding is in some ways generic and not necessarily directly unique to adolescent MRB outcomes, we nevertheless see it as important due to our programme theory that states that social environmental change is needed to reduce MRB, which is more complex, long-term and expensive than educational programmes [22].

Further, we were unable to identify particular *programmes* within the community mobilisation interventions that were noted as crucial (e.g. a school-based initiative or community component). While this was not an objective of our study due to our focus being on the wider community mobilisation and coalition effort, it would have no doubt been informative to communities hoping to implement these strategies. This absence of specific recommendations around programmes or components is partly due to the lack of description of said individual programmes, but also if we were to recommend specific programmes, we would be contradicting the impetus of community mobilisation interventions. That is, several studies suggested that success does not lie within the single components of the programmes delivered [20], but instead within the shift within society towards a way of thinking that fosters adolescent MRB prevention. This shift is more probable with comprehensive approaches, with a greater number of prevention activities at different levels [20]. However, we do recognise critiques that suggest that without identifying what the successful intervention components are, it is difficult to establish whether the intervention has had a positive impact on outcomes [91].

The composition of the coalition was highlighted as a key factor. Those studies with the greatest success strongly recommend a mixture of experts, policy stakeholders and community members, with ‘intervention champions’ being employed to maintain those connections [20, 22, 59, 63, 72, 74]. We identified that most of the coalitions were made up of adults only or did not report ways in which young people input into the intervention. The Australian version of Communities that Care adapted the original CTC model and created an active youth board who were involved in the ongoing development of the intervention [63]. This might be an indication of the time period of the studies, with many studies being designed before 2001. It was around this time that there was an acceleration in calls for participatory approaches to be adopted in public health research and practice [94]. The value of public involvement in public health intervention research is now well recognised [95]. Further, involving youth in the design and delivery of youth-based interventions is a matter of equity, social justice and reducing power imbalances [96]. The involvement of the target group, adolescents, has also been shown to be critical for intervention effectiveness [97]. We would anticipate that if community mobilisation interventions are to be successful in the future, the studies included in this review would need to be revised to incorporate greater youth involvement.

We were unable to identify CMOs related to the ‘for whom’ question in our synthesis. One document [83] reported gender differences in intervention effects, but could only offer a speculative explanation as to why there were greater reductions in boys’ health risk behaviours than girls. Across documents, there was often a lack of detail about the young people and community members who were the target of the intervention, with a greater focus on coalition members. Some studies described the intervention setting, in a general sense, in that the focus was on youth of colour [80] or from deprived areas [54]. Without a subgroup analysis or narrative reflection on how aspects of the intervention affected such groups differently, we were unable to draw any conclusions on any dimension of inequality. This finding is unsurprising given the period during which many of the studies were undertaken (1980s–2000s). The two previous systematic reviews on individual, family and school-level interventions for adolescent multiple health risk behaviour similarly found a lack of reporting of the demographic characteristics needed to assess differential intervention effects, particularly for older studies [6].

### Strengths and limitations

Realist reviews, like traditional systematic reviews, are ultimately limited by the data they can extract. The kind of information we needed to understand the contexts and mechanisms, such as process evaluation data, researcher reflections and challenges and successes are less likely to be published in peer-reviewed journals, if at all. The stakeholder engagement sessions provided some insight into the mechanisms and contexts surrounding some interventions. However, given the period that many of the interventions took place and how complex many of them were, we were limited in how much detail we could gather. Documents were also limited in the detail they provided around the composition of the coalition, the strategies they implemented and the population they reached. These data limitations also meant we were unable to draw any conclusions about health inequalities. Further, we know of several community mobilisation interventions that are planned or ongoing [87, 88, 93], but as they did not publish a report of outcomes, we could not include them in the analysis—although they were used to understand the patterns found within the included studies.

There may have been some interventions that were not picked up in our searches. Multiple risk behaviour is a construct referring to the co-occurrence of two or more health risk behaviours. However, this term is not widely used within the vast field of community mobilisation approaches. Given that our search terms included health risk behaviour, we may have missed some interventions that do not overtly mention health risk behaviour but measured relevant outcomes. Many interventions appeared relevant but were excluded because they only reported one health risk behaviour outcome. Further, the majority of programmes in our sample were delivered in the USA. Our searching style and syntax choices (e.g. choosing to restrict to ‘coalition-based interventions’) will have likely had an impact on our results. For instance, ‘coalition’ as a term may not be used commonly in contemporary research or outside of the USA—as is the case within the Icelandic example, which proved to be a key study not picked up by initial searches. Despite engagement with stakeholders outside of the USA, many studies related to the Communities that Care programme. This undoubtedly narrowed our analysis, particularly in the early middle range theory development stages.

## Conclusions

For community mobilisation interventions to ‘work’ and reduce adolescent multiple risk behaviour within the community, the coalitions within them must seek to alter the social environment in which these behaviours are likely to occur. Mechanisms including empowerment, motivation and knowledge lead to this success, but only under certain contexts. In particular, we found successful interventions that aligned with our middle range theories tended to (1) be guided by principles that were interwoven across the community, (2) establish community readiness and use population level data to make coalition decisions and (3) consider carefully the composition of the coalition so that the right balance of expertise and local knowledge were included to achieve social environmental change.

### Supplementary Information

**Additional file 1.** Programme Theory Evolution. The file provides a description of how the programme theory model was created in collaboration with stakeholders and reviewing the initial documents and how that model evolved to the final version.

**Additional file 2.** Realist review search strategy. The file contains a table with the syntax and search strategies for each database used for the systematic searching phase.

**Additional file 3.** Quality assessment and extraction form. The file contains the blank form used to assess the relevance and rigour of each document and extract the data from the documents.

**Additional file 4.** Documents and interventions included in review. The file contains the blank form used to assess the relevance and rigour of each document and extract the data from the documents.

## Data Availability

The search strategy and realist review process are available within the manuscript or the additional files. The documents used to extract data on the interventions are available online via their listed citations.

## Abbreviations

MRB: Multiple risk behaviour
NEET: Not being in education, employment or training
HIV: Human immunodeficiency virus
RCTs: Randomised controlled trials
CTC: Communities that Care
CMO: Context, Mechanism, Outcome
RAMESES: Realist and meta-narrative evidence syntheses: evolving standards
USA: United States of America
UK: United Kingdom
EBP: Evidence-based programmes
