# Supplementary material for: Community mobilisation approaches to preventing adolescent multiple risk behaviour: a realist review
Source: Syst Rev. 2024 Feb 26;13:75. doi: 10.1186/s13643-024-02450-2 (PMC10895861; doi:10.1186/s13643-024-02450-2)
Supplement: Supplementary file 1 — Additional file 1. Programme Theory Evolution. The file provides a description of how the programme theory model was created in collaboration with stakeholders and reviewing the initial documents and how that model evolved to the final version. [file 13643_2024_2450_MOESM1_ESM.docx]

One way the programme theory model altered as a result of discussion with stakeholders relates to the importance of implementing ‘environmental’ versus ‘individual-level’ programmes to young people. Two stakeholders emphasized that it is the **coalition and the health prevention system that is the intervention**, not the programmes the coalition choose to deliver. The chosen programmes should be evidence-based and coalition members are trained and encouraged to deliver a range of different programmes, with information about the potential pitfalls of only including educational programmes. However, the extent to which coalition implement certain types of programmes is not the underlying mechanism that leads to positive outcomes. Having a diverse and proactive coalition that embeds adolescent health risk behaviour prevention within local public health, through community champions or key leaders, is the underlying mechanism here. Therefore, the model altered to emphasize the coalition and prevention system as the intervention, without a measure of success being whether coalitions choose potentially more effective and equitable environmental approaches.

Inclusion of **community champions** as a key mechanistic factor driving the success of community mobilization interventions. This goes beyond employed co-ordinators and technical assistance. These champions are likely members of the coalition who communicate with local authority staff, school staff and local community members to embed the intervention within the consciousness of the wider community. This minimizes issues and delays as knowledge and support for the intervention is widespread before programmes are delivered or surveys are circulated.
