# Supplementary material for: Community mobilisation approaches to preventing adolescent multiple risk behaviour: a realist review
Source: Syst Rev. 2024 Feb 26;13:75. doi: 10.1186/s13643-024-02450-2 (PMC10895861; doi:10.1186/s13643-024-02450-2)
Supplement: Supplementary file 2 — Additional file 2. Realist review search strategy. The file contains a table with the syntax and search strategies for each database used for the systematic searching phase. [file 13643_2024_2450_MOESM2_ESM.docx]

# Systematic search strategy for realist review

We selected search terms from three categories: community terms, adolescent population terms, multiple risk behaviour terms. We combined these in each database.

| **Database** | **Community mobilization terms** | **AND Adolescent terms** | **AND multiple risk behaviour terms** |
| --- | --- | --- | --- |
| Medline | (Community mobilization or community networks or community groups or Community coalition or community mobilisation or community empowerment).mp.  Community Networks/  Community Participation/ | (Adolescen* or teen* or young person or young people or youth* or hooligan or young adult* or early adult* or juvenile* or minor? or emerging adult* or girl or boy or apprentice* or FE college* or young m#n or young wom#n or young male* or young female* or under 18* or sixth-form* or secondary education or tertiary education or higher education or further education).mp.  child/ or adolescent/ | ("Health risk behavio?r*" or "multiple risk behavio?r*" or "high risk behavio?r*" or "multiple risk factor*" or "behavio?r* risk factor*").mp. [mp=title, abstract, original title, name of substance word, subject heading word, protocol supplementary concept, rare disease supplementary concept, unique identifier] |
| PubMed | (((community mobilisation) OR (COMMUNITY MOBILIZATION) OR (community mobilization) OR (mobilizing community resources) OR (community coalition) ) OR (community coalitions) OR (community health coalition) OR (community coalition AND health promotion)) OR (MH community participation) | ((Adolescen* or teen* or "young person" or "young people" or youth* or hooligan* or "young adult*" or "early adult*" or juvenile* or minor or "emerging adult*" or girl or boy or apprentice* or "FE college*" or "young m?n" or "young male*" or "young female*" or "under 18*" or "sixth‐form*" or "secondary education" or "tertiary education" or "further education")) | (("smoke"[All Fields] OR "smoke s"[All Fields] OR "smoked"[All Fields] OR "smokes"[All Fields] OR "smoking"[MeSH Terms] OR "smoking"[All Fields] OR "smokings"[All Fields] OR "smoking s"[All Fields])) AND ((("alcohol*"[All Fields] OR ("ethanol"[MeSH Terms] OR "ethanol"[All Fields] OR "ethanols"[All Fields] OR "ethanol s"[All Fields] OR "ethanolic"[All Fields]) OR ("beer"[MeSH Terms] OR "beer"[All Fields]) OR ("cider"[All Fields] OR "ciders"[All Fields]) OR ("wine"[MeSH Terms] OR "wine"[All Fields]) OR "spirit*"[All Fields] OR "alcopop*"[All Fields]) AND "near 3"[All Fields]) AND ("use"[All Fields] OR "usage*"[All Fields])) AND (("substance*"[All Fields] AND "near 2"[All Fields]) AND ("abus*"[All Fields] OR "addict*"[All Fields] OR "depend*"[All Fields] OR "misus*"[All Fields] OR "use"[All Fields] OR ("usage"[All Fields] OR "usages"[All Fields]))) OR "gambl*"[All Fields] OR ("gambling"[MeSH Terms] OR "gambling"[All Fields] OR "betting"[All Fields]) OR (("risky sex*" OR "unsafe sex*" OR "safe* sex*" OR "sexual intercourses" OR "reproductive behavio*" OR "sexual behavio*" OR "sexual health") OR (contracepti* OR condom OR condoms OR "morning after pill*")) OR (("sexually transmitted disease*" OR "sexually transmitted infection*" OR STD* OR STI OR sits OR chlamydia* OR gonorrhoea* OR HIV OR AIDS OR "human immunodeficiency virus" OR "acquired immunodeficiency syndrome")) OR ((delinquenc* OR delinquent* OR offen* OR reoffend* OR violen* OR theft* OR robbery OR burglary* OR steal* OR "criminal damage" OR joyrid* OR "joy rid*" OR assault* OR (sell* NEAR/1 drug*) OR devian* OR "anti social behavio*" OR "antisocial behavio*" OR graffiti OR "index crime*" OR (breaking NEAR/1 entering) OR "strong‐arming" OR "pan‐handling" OR panhandling OR "disorderly conduct" OR prostitut* OR (carry* NEAR/2 weapon*) OR (buy* N2 stolen) OR "criminal behavio*" OR (noisy NEAR/1 rude) OR (nuisance* NEAR/2 neighbo*) OR fight* OR stab? OR stabbing OR stabbed OR stabwound* OR wound* OR aggress* OR weapon* OR knife* OR knives OR gun* OR firearm* OR murder*) OR (bicyc* OR cycl* OR bik* OR motorbike* OR motorcycl*)) OR (selfharm* OR "physical activity" OR nutrition OR diet*) OR ( ( (suicid* or harm* or injur* or hurt*) n2 (gesture* or behavio#r*) ) OR ( ("para suicid*" or parasuicid* or "attempt* suicid*" ) ) OR ( ("non fatal" or nonfatal) n2 (suicid* or harm*) ) ) OR ( (MH "Self‐Injurious Behavior") OR (MH "Injuries, Self‐Inflicted") ) OR ( (calori* or fat or fatty or fizz* or soft* or carbonated* or sweetened or salt* or sugar* or fruit* or veg or vegetable* or fibre* or fiber* or "5‐a‐day" or "five a day") N2 (intake or food* or diet* or consum* or meal* or eat* or nutrition or drink* or snack*) ) OR ( (poor or over* or unhealthy or health*) N3 (nutrition or diet* or eat* or meal* or food* or snack* or drink*) ) OR ( (MH "Physical Activity") OR (MH "Physical Fitness") ) OR ( "Physical activity" or fitness or "physical* fit*" or "physical exert*" or exercise or "aerobic activit*" or sport* or "aerobic capacity" or "active lifestyle*" or "outdoor activit*" or gym* or mvpa ) OR ( ( (screen or sedentary or view*) N2 (time or hour* or minute*) ) OR ( (inactiv* or seden* or indoor*) N2 (lifestyle* or activit*) ) ) |
| PsycINFO | (Community mobilization or community networks or community groups or Community coalition or community mobilisation or community empowerment).mp.  Community Networks/  Community Participation/ | (Adolescen* or teen* or young person or young people or youth* or hooligan or young adult* or early adult* or juvenile* or minor? or emerging adult* or girl or boy or apprentice* or FE college* or young m#n or young wom#n or young male* or young female* or under 18* or sixth-form* or secondary education or tertiary education or higher education or further education).mp.  child/ or adolescent/ | ("Health risk behavio?r*" or "multiple risk behavio?r*" or "high risk behavio?r*" or "multiple risk factor*" or "behavio?r* risk factor*").mp. [mp=title, abstract, original title, name of substance word, subject heading word, protocol supplementary concept, rare disease supplementary concept, unique identifier] |
| Web of Science |  | (Adolescen* OR teen* or young person or young people or youth* or hooligan or young adult* or early adult* or juvenile* or minor or emerging adult or girl or boy or apprentice or FE college or young men or young women or young male or young female or under 18 or sixth-form* or secondary education or tertiary education or higher education or further education) | ( health risk behaviour* OR health risk behavior* OR multiple risk behaviour* OR multiple risk behavior* OR "high risk behaviour* OR high risk behavior* OR multiple risk factor* OR "behavior* risk factor* OR behavior* risk factor*)  TS= (substance* OR addict* OR gambl* OR betting OR risky sex* OR unsafe sex* OR safe* sex* OR sexual intercourses OR reproductive behavior* OR reproductive behaviour* OR sexual behavior* OR sexual behaviour* OR sexual health OR contracepti* OR condom OR condoms OR morning after pill* OR sexually transmitted disease* OR sexually transmitted infection* OR STD* OR STI OR sits OR chlamydia* OR gonorrhea*OR OR HIV OR AIDS OR human immunodeficiency virus OR acquired immunodeficiency syndrome)  TS= (smoking OR alcohol* OR ethanol OR beer OR cider OR wine OR spirit* OR alcopop*)  TS= (tobacco OR cigarette* OR nicotine)  TS= (smoking OR alcohol* OR ethanol OR beer OR cider OR wine OR spirit* OR alcopop*)  TS= (delinquenc* OR delinquent* OR offen* OR reoffend* OR violen* OR theft* OR robbery OR burglary* OR steal* OR criminal damage OR joyrid* OR joy rid* OR assault*) |
| CINAHL | Community mobilization or community networks or community groups or Community coalition or community mobilisation or community empowerment | (Adolescen* or teen* or “young person” or “young people” or youth* or hooligan* or “young adult*” or “early adult*” or juvenile* or minor or “emerging adult*” or girl or boy or apprentice* or “FE college*” or “young m?n” or “young wom?n” or “young male*” or “young female*” or “under 18*” or “sixth‐form*” or “secondary education” or “tertiary education” or “further education”) | ( ( ( ( ( "health risk behavio#r*" OR "multiple risk behavio#r*" OR "high risk behavio#r*" OR "multiple risk factor*" OR "behavio#r* risk factor*" ) OR (MH "Risk Taking Behavior+") OR (MH "Smoking") OR (MH "Alcohol Drinking") OR (MH "Substance Use Disorders") OR ( ( (marijuana or cannabis or "recreational drug*" or "class c"or "white widow*") ) N2 ( (abus* or use* or using or usage or misus* or smok* or addict* or depend*) ) ) OR ( substance* N2 (abus* or addict* or depend* or inject* or intravenous or misus* or use* or usage or using) ) OR ( ("class a" or "class b" or drug* or cocaine or ecstasy or mdma or glue or gas or aerosol* or solvent* or "magic mushroom*" or crack or ketamine or heroin or morphine or narcotic* or opiat* or opiod* or popper* or lsd* or methamphetamine* or amphetamine*) N2 (abus* or addict* or depend* or inhal* or misus* or sniff* or use* or usage or using) ) OR ( ( tobacco or cigarette* or nicotine ) N3 ( addict* or use* or usage or using or intake or consum*) ) OR ( ( (alcohol* or ethanol or beer or cider or wine or spirit* or alcopop*) ) N3 ( (use* or usage* or using or intake or consum* or drink* or misus* or abus*) ) ) OR ( ( (alcohol* or drink* or ethanol) ) N3 ( (excess* or binge* or binging or intoxicat* or poison* or risk* or depend*) ) ) ) OR ( gambl* or betting ) OR ( ( "risky sex*" or "unsafe sex*" or "safe* sex*" ) OR ( "sexual intercourse" or "reproductive behavio#r*" ) OR ( "sexual behavio#r*" or "sexual health" ) ) OR ( ( delinquen* or offen* or reoffend* or violen* or theft* or robbery or burglar* or steal* ) OR ( "criminal damage" or joyrid* or "joy rid*" or assault* ) OR ( "sell* N1 drug*" or "devian*" or "anti social behavio#r*" or " antisocial behavio#r*" ) OR ( graffiti or "racist abuse" or "index crime*" or (breaking N1 entering) or "strong arming" ) ) ) OR ( ( "pan handling" or panhandling or "disorderly conduct" or prostitut* or (carry* N2 weapon*) or (buy* N2 stolen) or "criminal behavio#r" or (noisy N1 rude) or (nuisance* N2 neighbo#r*) ) OR ( fight* or stab or stabbing or stabbed or stabwound* or wound* or aggress* or weapon* ) OR ( knife or knives or gun* or firearm* or murder* ) ) OR ( ( ((youth* or street or criminal or adolescen* or juvenile* or teen*) N2 gang*) ) OR ( (adolescen* or youth* or juvenile* or delinquen* or teen* or gang or school* or college* or "sixth form*" ) N2 (crim* or offen* or violen* or fight*) ) ) OR ( ( (use or using or usage or wear* or wore) N2 ("seat belt*" or seatbelt* or "safety belt*") ) OR injur* N2 behav* OR ( (alcohol* or intoxica* or dr?nk*) N2 (driv* or vehicle* or motor* or car# or van# or automobile* or "auto mobile*") ) ) OR ( (bicyc* or cycl* or bik* or motorbike* or motorcycl*) N2 (helmet* or protect* or "risk reduc*" or "head gear") N2 (lack or without or absen* or wear* or no or non#) ) ) OR ( ( (suicid* or harm* or injur* or hurt*) n2 (gesture* or behavio#r*) ) OR ( ("para suicid*" or parasuicid* or "attempt* suicid*" or "suicid* attempt*") ) OR ( ("non fatal" or nonfatal) n2 (suicid* or harm*) ) ) OR ( (MH "Self‐Injurious Behavior") OR (MH "Injuries, Self‐Inflicted") ) ) OR ( (calori* or fat or fatty or fizz* or soft* or carbonated* or sweetened or salt* or sugar* or fruit* or veg or vegetable* or fibre* or fiber* or “5‐a‐day” or “five a day” or “go for 2&5”) N2 (intake or food* or diet* or consum* or meal* or eat* or nutrition or drink* or snack*) ) OR ( (poor or over* or unhealthy or health*) N3 (nutrition or diet* or eat* or meal* or food* or snack* or drink*) ) OR ( (MH "Physical Activity") OR (MH "Physical Fitness") ) OR ( “Physical activity” or fitness or “physical* fit*” or ”physical exert*” or exercise or “aerobic activit*” or sport* or “aerobic capacity” or “active lifestyle*” or “outdoor activit*” or gym* or mvpa ) OR (MH "Life Style, Sedentary") OR ( ( (screen or sedentary or view*) N2 (time or hour* or minute*) ) OR ( (inactiv* or seden* or indoor*) N2 (lifestyle* or activit*) ) ) |
| Sociological Abstracts | ((community mobilisation) OR (COMMUNITY MOBILIZATION) OR (community mobilization) OR (mobilizing community resources) OR (community coalition) ) OR (community coalitions) OR (community health coalition) OR (community coalitions and health promotion) | all(Adolescen* OR teen* OR “young person” OR “young people” OR youth* OR hooligan* OR “young adult*” OR “early adult*” OR juvenile* OR minor OR “emerging adult*” OR girl OR boy OR apprentice* OR “FE college*” OR “young m*n” OR “young wom*” OR “young male*”or “young female*” OR “under 18*” OR “sixth‐form*” OR “secondary education” OR “tertiary education” OR “higher education” OR “further education”) | all("Health risk behavio*" OR "multiple risk behavio*" OR "high risk behavio*" OR "multiple risk factor*" OR "behavio* risk factor*") OR all((tobacco OR cigarette* OR nicotine) NEAR/3 (addict* OR use* OR usage OR using OR intake OR consum*)) OR all(smoking) OR all((alcohol* OR ethanol OR beer OR cider OR wine OR spirit* OR alcopop*) NEAR/3 (use* OR usage*)) OR all(substance* NEAR/2 (abus* OR addict* OR depend* OR misus* OR use* OR usage OR using)) OR gambl* OR betting OR all("risky sex*" OR "unsafe sex*" OR "safe* sex*" OR "sexual intercourses" OR "reproductive behavio*" OR "sexual behavio*" OR "sexual health") OR all(contracepti* OR condom OR condoms OR "morning after pill*") OR all("sexually transmitted disease*" OR "sexually transmitted infection*" OR STD* OR STI OR sits OR chlamydia* OR gonorrhea*OR gonorrhoea* OR HIV OR AIDS OR "human immunodeficiency virus" OR "acquired immunodeficiency syndrome") OR all (delinquenc* OR delinquent* OR offen* OR reoffend* OR violen* OR theft* OR robbery OR burglary* OR steal* OR "criminal damage" OR joyrid* OR "joy rid*" OR assault* OR (sell* NEAR/1 drug*) OR devian* OR "anti social behavio*" OR "antisocial behavio*" OR graffiti OR "racism abuse" OR "index crime*" OR (breaking NEAR/1 entering) OR "strong‐arming" OR "pan‐handling" OR panhandling OR "disorderly conduct" OR prostitut* OR (carry* NEAR/2 weapon*) OR (buy* N2 stolen) OR "criminal behavio*" OR (noisy NEAR/1 rude) OR (nuisance* NEAR/2 neighbo*) OR fight* OR stab? OR stabbing OR stabbed OR stabwound* OR wound* OR aggress* OR weapon* OR knife* OR knives OR gun* OR firearm* OR murder*) OR all(bicyc* OR cycl* OR bik* OR motorbike* OR motorcycl*) AND all((helmet* OR protect* OR "risk reduc*" OR "head gear" OR "head protection") NEAR/3 (lack* OR no OR no? OR without OR absen* OR wear*)) OR selfharm* OR "physical activity" OR nutrition OR diet*) |
