# Supplementary material for: Community mobilisation approaches to preventing adolescent multiple risk behaviour: a realist review
Source: Syst Rev. 2024 Feb 26;13:75. doi: 10.1186/s13643-024-02450-2 (PMC10895861; doi:10.1186/s13643-024-02450-2)
Supplement: Supplementary file 3 — Additional file 3. Quality assessment and extraction form. The file contains the blank form used to assess the relevance and rigour of each document and extract the data from the documents. [file 13643_2024_2450_MOESM3_ESM.docx]

## Quality Assessment and Data Extraction Form

**How, why, for whom and in what circumstances and time periods’ do community mobilisation interventions work to prevent and/or reduce adolescent multiple risk behaviour?**

**Quality Assessment and Data Extraction Form for Full Articles:**

| **Question** | **Options** | **Definitions/Additional notes** |
| --- | --- | --- |
| **Relevance Verification** | | |
| What language is the article in? | - English - Other (exclude) |  |
| Does this citation describe **community mobilization** interventions targeting **two or more** adolescent health risk behaviours? | - Yes – relevant research - Yes – relevant but does not measure behaviour outcomes - Yes – relevant but does not target **two** health risk behaviors (exclude) - Yes – relevant but does not describe community mobilisation intervention (e.g. coalition) (exclude) - No - not relevant (exclude) - No – but may be useful for further reading (exclude) - No – but check that we have included citations from this article (exclude) | **Interventions:** a program or strategy designed to produce behavior changes or improve health among individuals or populations. In this instance, the ‘community mobilisation’ or coalition formation element is the intervention. The coalition then implement their own strategies within the wider intervention.  **Behaviours:** alcohol, tobacco, cannabis, illicit drugs, anti-social behaviour, risky sexual behaviour, physical inactivity, self-harm, vehicle risk behaviours. Others may be included but at least two of these should be the target (not necessarily measured as an outcome yet). |
| Specify all **behaviours** that this community mobilisation effort is targeting. | - Smoking cessation - Alcohol consumption - Cannabis - Illicit drug use - Physical activity/physical inactivity - Risky sexual behaviour - Self harm - Anti-social behaviour - Vehicle risk - Health promoting behaviours - Other (please state) |  |
| **If exclusion criteria were selected above, submit the form before proceeding** | | |
| **Data Extraction** | | |
| **General Information** | | |
| What year was the article published? | [text] |  |
| What type of document is this article? | - Primary research - Editorial/commentary/protocol/ challenges - Thesis - Conference proceeding - Literature review - Systematic review/meta-analyses - Book - Grey literature | **Primary research:** original research/investigation/study carried out by the researcher (includes surveys, interviews, outbreak reports, observations etc.)  **Editorial/commentary/protocol/ challenges:** The paper may be referring to original research or a community mobilisation intervention, but the purpose of the document is to outline the steps taken to achieve the intervention or discuss the challenges in doing so.  **Thesis:** a long paper/essay or dissertation involving personal research (usually written for a university degree)  **Conference proceeding abstract/short paper:** an individual or collection of published academic papers  **Literature review:** examination of published literature  **Systematic review/meta-analyses:** analysis and interpretation of primary research  **Grey literature:** research that is unpublished or published in a non-commercial form (e.g. newspaper or magazine articles) |
| On what continent did the intervention take place?  (If not specified, resort to authour affiliations)  Specify the country, state or province. | - Europe (UK?) - USA - Australasia - Central America/South America/Caribbean - Asia - Africa - Other: [text]   [text] | **North America:** includes Canada, USA and Mexico  **Europe:** includes, Belarus, Latvia, Ukraine, Estonia, Cyprus & west (includes Iceland and Greenland)  **Australasia:** limited to Australia, New Guinea, New Zealand, New Caledonia, and neighbouring islands, including the Indonesian islands from Lombok and Sulawesi eastward  **Central America/South America/ Caribbean:** includes Caribbean, and all of south and central America.  **Asia:** Russia, Turkey, middle eastern countries and east |
| What is the study design? | - Observational study - Cohort - Case-control - Other, specify: [text] - Experimental study - Randomized control trial - Quasi-experimental study - Other, specify: [text] - Qualitative study | **Cohort:** follow a group of exposed and non-exposed individuals to evaluate whether they develop an outcome  **Case control:** identified cases are matched with controls and their risk factors are evaluated for an association with outcome  **Randomized Control Trial:** individuals are allocated at random to a control or intervention group  **Quasi-experiment:** used to estimate the causal impact of an intervention on its target population without random assignment  **Qualitative:** primarily exploratory research. It is used to gain an understanding of underlying reasons, opinions, and motivations (e.g. focus groups, interviews etc.) |
| Briefly describe the intervention | [text] |  |
| **Coalition** | | |
| How many people are on the coalition? | Yes  No  Not stated |  |
| Was the coalition newly formed for the intervention? | Yes  No  Not stated |  |
| Does the coalition involve strategic ‘leaders’? | Yes, fully  Yes, partly  No  Not stated | **Strategic Leaders:** Individuals with a level of local, regional or national power. They are involved (as part of their employment) in activities related to education, policing, health etc. They may be community residents, but they should be primarily have been chosen for their strategic input. |
| Does the coalition involve ‘community leaders’? | Yes, fully  Yes, partly  No  Not stated | **Community leaders:** Individuals within the community who have a strong interest in community improvement and adolescent health, but are not necessarily involved in local decision making and policies in the same way that strategic leaders are. They may be religious leaders, school leaders, business owners or community residents. |
| Are young people involved in the coalition? | Yes, on the main coalition  Yes, as a youth advisory panel  No |  |
| Is there an oversight body that the coalition report to? | Yes  No  Not stated |  |
| Do the coalition receive paid technical assistance? | Yes  No  Not stated | **Technical assistance:** This may be academics or trained personnel who guide the coalition through the community mobilisation process |
| Does the coalition receive funding? | Yes  No |  |
| **Target Population (young people and community residents)** | | |
| What is the sample size of participants in the study? (Specify for both intervention and control) | - Total [text] - Intervention [text] - Control [text] - Not stated |  |
| Indicate the number of male and female participants in the study. (Specify for both intervention and control) | - Male [text] - Female [text] - Not stated |  |
| What was the target population of this intervention? *(Check all that apply)* | - General public (community residents) - Families - Young people - Students in schools - Minority groups, specify: [text] - Disadvantaged, specify: [text] - Other, specify: [text] | Specify any relevant details about the target population |
| What age range are the participants of the study? (Specify for both intervention and control) |  |  |
| What is the race of the participants in the study? *(Specify for both intervention and control)* | - White/Caucasian [text] - Black or African American [text] - Aboriginal or Indigenous [text] - Asian/Pacific Islander [text] - Hispanic or Latino [text] - Other, specify: [text] - Not stated |  |
| Was any additional demographic information about the study participants captured? *(Specify for both intervention and control)* | - Socioeconomic status [text] - Occupation [text] - Education [text] - Culture/ethnicity [text] - Other, specify: [text] - Not stated |  |
| **Context** | | |
| Were any contextual factors discussed? | - Infrastructure [text] - Characteristics of region [text] - Social environment (prevailing norms, membership groups, culture) - Physical environment (financial resources, material resources, time pressures, location) - Other, specify: [text] - Not stated | **Infrastructure:** E.g. having an existing multi-disciplinary infrastructure (access to nurses, dietitians etc.) that the intervention can leverage  **Characteristics of region:** established evidence-base for severity of health outcomes in the region of intervention |
| **Activities** | | |
| What types of activities did the intervention use? |  | List all that apply |
| **Mechanisms** | | |
| Were any mechanisms identified or inferred? | - Resource mechanism - Reasoning mechanism | List all that apply |
| **Outcomes** | | |
| Describe the impact of the intervention. | - Multiple risk behaviour outcomes [text] - Intermediate outcomes [text] - Implementation/process outcomes [text] - Other biological impacts [text] |  |
| How were the outcomes measured? | [text] |  |
| Describe any facilitators or barriers to the success of the intervention/program | [text] | E.g. engagement of participants |
| **Quality Assessment** | | |
| What type of study was this? | - Qualitative (move to qualitative questions) - Quantitative (move to quantitative questions) - Mixed-Method (move to mixed-method questions) | **Qualitative:** research that gathers information that is not numerical (e.g. interviews, focus groups etc.)  **Quantitative:** research that gathers numerical data that can be put into categories, or in rank order, or measured in units of measurement  **Mixed-method:** research that involves the mixing of quantitative and qualitative methods (e.g. exploratory design) |
| **Qualitative Quality Assessment** | | |
| Was there a clear statement of the research purpose/aims? | - Yes [text] - No [text] - Can’t Tell [text] | Consider the following to make a judgement: clarity of focus, explicit purpose given, supported by prior research |
| Does the collected data address the research question (or objectives)? | - Yes [text] - No [text] - Can’t Tell [text] | Consider whether the research and data collection strategy clearly described and was appropriate to address the research or objectives |
| Are the sources of qualitative data relevant to address the research question (objective)? | - Yes [text] - No [text] - Can’t Tell [text] | *Was the recruitment strategy appropriate to the aims of the research?* Consider whether (a) the selection of the participants is clear and appropriate to collect relevant and rich data; and (b) reasons why certain potential participants were chosen, or chose not to participate |
| Is the process for analyzing qualitative data relevant to address the research question (objective)? | - Yes [text] - No [text] - Can’t Tell [text] | *Was the data collected in a way that addressed the research issue?* Consider whether (a) the method of data collection is clear (e.g. focus group, interview); (b) the form of the data is clear (e.g. tape recordings, video material); (c) changes are explained when methods are altered during the study; (d) the qualitative data analysis addresses the question; and (e) the researcher has discussed saturation of data |
| Is appropriate consideration given to how findings relate to the context, e.g., the setting in which the data were collected? | - Yes [text] - No [text] - Can’t Tell [text] | Consider whether the study context and how findings relate to the context and characteristics of the context are explained (how findings are influenced by or influence the context) |
| Is appropriate consideration given to how findings relate to researchers’ influence, e.g., through their interactions with participants? | - Yes [text] - No [text] - Can’t Tell [text] | *Has the relationship between researcher and participants been adequately considered?* Consider whether (a) researchers critically explain how findings relate to their perspective, role, and interactions with participants (how the research process is influenced); (b) researcher’s role is influential at all stages; and (c) researchers explain their reaction to critical events that occurred during the study |
| Was the data analysis sufficiently rigorous? | - Yes [text] - No [text] - Can’t Tell [text] | Consider whether (a) data provided sufficient depth, detail and richness? (e.g. illustrative quotes); (b) an in-depth description of the analysis process (c) context described and taken into account in interpretation/results; (d) approaches taken to ensure robustness (e.g. multiple analysts, triangulation, member checking/participant validation of results) |
| Have ethical issues been taken into consideration? | - Yes [text] - No [text] - Can’t Tell [text] | Consider whether (a) study was approved by ethics committee; (b) sufficient details provided on how the research was explained to participants and whether ethical standards were maintained; (c) documentation of how autonomy, consent, confidentiality, anonymity were managed; and (d) documentation of any ethical dilemmas and how they were resolved |
| Is there a clear statement of findings? | - Yes [text] - No [text] - Can’t Tell [text] | Consider whether (a) the findings are explicit; (b) adequate discussion of the evidence both for and against the researchers arguments; (c) researcher has discussed the credibility of their findings (e.g. triangulations, respondent validations); and (d) findings are discussed in relation to research question |
| Was there evidence of study relevance and transferability? | - Yes [text] - No [text] - Can’t Tell [text] | Consider whether (a) there is a discussion of contribution of study to existing/prior knowledge, practice, and/or policy; (b) areas for future research identified; (c) limitations/weaknesses of study are clearly outlined; and (d) there is a discussion of whether or how the findings can be transferred to other populations or consideration of other ways the research may be used |
| **Quantitative Quality Assessment** | | |
| Was the study a randomized control trial? | - Yes (moves to randomized control questions) - No (moves to non-randomized study questions) | **Randomized Control Trial:** individuals are allocated at random to a control or intervention group |
| *Randomized Control Trials* | | |
| Was there a clear statement of the research purpose/aims? | - Yes [text] - No [text] - Can’t Tell [text] | Consider the following to make a judgement: clarity of focus, explicit purpose given, supported by prior research |
| Does the collected data address the research question (or objectives)? | - Yes [text] - No [text] - Can’t Tell [text] | Consider whether the research and data collection strategy clearly described and was appropriate to address the research or objectives |
| Is there a clear description of the randomization (or appropriate sequence generation)? | - Yes [text] - No [text] - Can’t Tell [text] | Consider whether researchers describe how the randomization or allocation is generated. A simple statement such as “we randomly allocated” or “using a randomized design” does not suffice. |
| Is there a clear description of the allocation concealment (or blinding when applicable)? | - Yes [text] - No [text] - Can’t Tell [text] - Not applicable | Consider whether (a) researchers and participants were unaware of the assignment sequence up to the point of allocation; or (b) researchers and participants are unaware of the group a participant is allocated to during the course of the study |
| Were the groups similar at the start of the trial? | - Yes [text] - No [text] - Can’t Tell [text] | Consider other factors that might affect the outcome such as age, sex, social class etc. |
| Are there complete outcome data (80% or above)? | - Yes [text] - No [text] - Can’t Tell [text] | Almost all of the participants contributed to almost all measures |
| Is there low withdrawal/drop-out (below 20%)? | - Yes [text] - No [text] - Can’t Tell [text] | Almost all of the participants completed the study |
| Was there evidence of study relevance and transferability? | - Yes [text] - No [text] - Can’t Tell [text] | Consider whether (a) there is a discussion of contribution of study to existing/prior knowledge, practice, and/or policy; (b) areas for future research identified; (c) limitations/weaknesses of study are clearly outlined; and (d) there is a discussion of whether or how the findings can be transferred to other populations or consideration of other ways the research may be used |
| *Non-Randomized Studies* | | |
| Was there a clear statement of the research purpose/aims? | - Yes [text] - No [text] - Can’t Tell [text] | Consider the following to make a judgement: clarity of focus, explicit purpose given, supported by prior research |
| Does the collected data address the research question (or objectives)? | - Yes [text] - No [text] - Can’t Tell [text] | Consider whether the research and data collection strategy clearly described and was appropriate to address the research or objectives |
| Are participants (organizations) recruited in a way that minimizes selection bias? | - Yes [text] - No [text] - Can’t Tell [text] | **Cohort:** consider whether the exposed (or with intervention) and non-exposed (or without intervention) groups are recruited from the same population  **Case-control:** consider whether (a) same inclusion and exclusion criteria were applied to cases and controls; (b) whether recruitment was done independently of the intervention or exposure status; and (c) was there a sufficient number of cases/controls selected?  **Cross-sectional:** consider whether the sample is representative of the population |
| Are measurements appropriate (clear origin, or validity known, or standard instrument; and absence of contamination between groups when appropriate) regarding the exposure/intervention and outcomes? | - Yes [text] - No [text] - Can’t Tell [text] | Consider whether (a) the variable are clearly defined and accurately measured; (b) the measurements are justified and appropriate for answering the research question, and (c) the measurements reflect what they are supposed to measure |
| In the groups being compared, are the participants comparable, or do researchers take into account (control for) the differences between the groups? | - Yes [text] - No [text] - Can’t Tell [text] | Have the authours taken account of the potential confounding factors in the design and/or analysis? Consider whether (a) the most important factors are taken into account in the analysis; (b) a table lists key demographic information comparing both groups, and there are no obvious dissimilarities between groups that may account for any differences in outcomes, or dissimilarities are taken into account in the analysis |
| Are there complete outcome data (80% or above), and, when applicable, an acceptable response rate (60% or above), or an acceptable follow-up rate for cohort studies (depending on the duration of follow-up)? | - Yes [text] - No [text] - Can’t Tell [text] |  |
| Did the authors report all outcomes? | - Yes [text] - No [text] - Can’t Tell [text] | Consider (a) if there is no evidence that outcomes were selectively reported (e.g. all relevant outcomes in the methods section are reported in the results section); (b) what are the bottom line results?; and (c) are the results adjusted for confounding, and might confounding still explain the association? |
| If a questionnaire was used to measure outcomes, was it appropriately validated and reliably tested? | - Yes [text] - No [text] - Can’t Tell [text] - Not applicable [text] | Consider if the questionnaire/focus group was appropriately validated and tested |
| Was there evidence of study relevance and transferability? | - Yes [text] - No [text] - Can’t Tell [text] | Consider whether (a) there is a discussion of contribution of study to existing/prior knowledge, practice, and/or policy; (b) areas for future research identified; (c) limitations/weaknesses of study are clearly outlined; and (d) there is a discussion of whether or how the findings can be transferred to other populations or consideration of other ways the research may be used |
| Was the study free of other problems that could put it at a high risk of bias? | - Yes [text] - No [text] - Can’t Tell [text] | Consider if you have any additional concerns about the design and/or conduct and reporting of this study |
| **Mixed-Method Quality Assessment** | | |
| Was there a clear statement of the research purpose/aims? | - Yes [text] - No [text] - Can’t Tell [text] | Consider the following to make a judgement: clarity of focus, explicit purpose given, supported by prior research |
| Does the collected data address the research question (or objectives)? | - Yes [text] - No [text] - Can’t Tell [text] | Consider whether the research and data collection strategy clearly described and was appropriate to address the research or objectives |
| Is the mixed methods research design relevant to address the qualitative and quantitative research questions (or objectives)? | - Yes [text] - No [text] - Can’t Tell [text] | Consider if the rationale for integrating qualitative and quantitative methods to answer the research question is explained. |
| Is the integration of qualitative and quantitative data (or results) relevant to address the research question (objective)? | - Yes [text] - No [text] - Can’t Tell [text] | There is evidence that data gathered by both research methods was brought together to form a complete picture, and answer the research question; authours explain when integration occurred; they explain how integration occurred and who participated in this integration |
| Is appropriate consideration given to the limitations associated with this integration, e.g., the divergence of qualitative and quantitative data (or results)? | - Yes [text] - No [text] - Can’t Tell [text] |  |
| Was there evidence of study relevance and transferability? | - Yes [text] - No [text] - Can’t Tell [text] | Consider whether (a) there is a discussion of contribution of study to existing/prior knowledge, practice, and/or policy; (b) areas for future research identified; (c) limitations/weaknesses of study are clearly outlined; and (d) there is a discussion of whether or how the findings can be transferred to other populations or consideration of other ways the research may be used |
| **Final Section** | | |
| Describe any important details that you believe were not extracted. | [text] |  |
