# Supplementary material for: Community mobilisation approaches to preventing adolescent multiple risk behaviour: a realist review
Source: Syst Rev. 2024 Feb 26;13:75. doi: 10.1186/s13643-024-02450-2 (PMC10895861; doi:10.1186/s13643-024-02450-2)
Supplement: Supplementary file 4 — Additional file 4. Documents and interventions included in review. The file contains the blank form used to assess the relevance and rigour of each document and extract the data from the documents. [file 13643_2024_2450_MOESM4_ESM.docx]

**Table 1: Number of included documents and outcomes of interest for each intervention**

| **Interventions (n=22)** | **Number of documents addressing types of outcomes** | | | **Total** |
| --- | --- | --- | --- | --- |
|  | **Health risk behaviour outcomes** | **Coalition functioning outcomes** | **Community level or intermediate outcomes (e.g., risk and protective factors)** |  |
| Icelandic Prevention Model (IPM) | 1 | 0 | 0 | 1 |
| Icelandic Prevention Model (IPM) implementation in Chile | 1 | 0 | 0 | 1 |
| Icelandic Prevention Model (IPM) implementation in Lithuania | 1 | 0 | 0 | 1 |
| Community Prevention and Wellness Initiative (CPWI) | 1 | 0 | 0 | 1 |
| Connect to Protect (C2P) Thailand | 1 | 0 | 0 | 1 |
| CSAP Community Partnership - Fighting Back | 1 | 0 | 0 | 1 |
| Communities that Care (CYDS) | 13 | 13 | 2 | 28 |
| Communities that Care (Pennsylvania) | 3 | 3 | 0 | 6 |
| Communities that Care (Australia) | 1 | 0 | 0 | 1 |
| Communities that Care (Netherlands) | 0 | 2 | 0 | 2 |
| Communities that Care (UK) | 0 | 2 | 0 | 2 |
| Communities that Care (Scottish Pilot) | 0 | 1 | 0 | 1 |
| TOGETHER! (CTC Oregon) | 0 | 2 | 0 | 2 |
| Kentucky Initiatives for Prevention (KIP) | 1 | 0 | 0 | 1 |
| New Directions | 1 | 0 | 0 | 1 |
| Project Freedom (Lawrence) | 1 | 0 | 0 | 1 |
| Project Freedom (Wichita) | 1 | 0 | 0 | 1 |
| PROSPER | 5 | 4 | 2 | 11 |
| Red de Coaliciones Comunitarias de Mexico (The Network of Community Coalitions in Mexico) | 0 | 1 | 0 | 1 |
| Minority Youth Health Project | 0 | 1 | 0 | 1 |
| The Youth Community Coalition (YC2) of Columbia | 0 | 2 | 0 | 2 |
| The Gloucester Prevention Network | 1 | 0 | 0 | 1 |
| Total | 30 | 33 | 4 | 69 |
